# A blood and bronchoalveolar lavage protein signature of rapid FEV_1_ decline in smoking-associated COPD

**DOI:** 10.1038/s41598-023-32216-0

**Published:** 2023-05-22

**Authors:** Katarina M. DiLillo, Katy C. Norman, Christine M. Freeman, Stephanie A. Christenson, Neil E. Alexis, Wayne H. Anderson, Igor Z. Barjaktarevic, R. Graham Barr, Alejandro P. Comellas, Eugene R. Bleecker, Richard C. Boucher, David J. Couper, Gerard J. Criner, Claire M. Doerschuk, J. Michael Wells, MeiLan K. Han, Eric A. Hoffman, Nadia N. Hansel, Annette T. Hastie, Robert J. Kaner, Jerry A. Krishnan, Wassim W. Labaki, Fernando J. Martinez, Deborah A. Meyers, Wanda K. O’Neal, Victor E. Ortega, Robert Paine, Stephen P. Peters, Prescott G. Woodruff, Christopher B. Cooper, Russell P. Bowler, Jeffrey L. Curtis, Kelly B. Arnold, SPIROMICS investigators

**Affiliations:** 1 Department of Biomedical Engineering, University of Michigan, Ann Arbor, MI USA; 2 Research Service, VA Ann Arbor Healthcare System, Ann Arbor, MI USA; 3 Division of Pulmonary & Critical Care Medicine, University of Michigan, Ann Arbor, MI USA; 4 Graduate Program in Immunology, University of Michigan, Ann Arbor, MI USA; 5 Division of Pulmonary, Critical Care, Allergy and Sleep Medicine, University of California San Francisco, San Francisco, CA USA; 6 Center for Environmental Medicine, Asthma, and Lung Biology, University of North Carolina at Chapel Hill, Chapel Hill, NC USA; 7 Marsico Lung Institute/Pulmonary and Critical Care Medicine, University of North Carolina at Chapel Hill, Chapel Hill, NC USA; 8 Division of Pulmonary and Critical Care Medicine, Department of Medicine, University of California Los Angeles, Los Angeles, CA USA; 9 Department of Medicine, Columbia University Medical Center, New York, NY USA; 10 Division of Pulmonary, Critical Care and Occupational Medicine, University of Iowa, Iowa City, IA USA; 11 Division of Genetics, Genomics and Precision Medicine, University of Arizona Health Sciences, Tucson, AZ USA; 12 Marsico Lung Institute/Cystic Fibrosis Research Center, Department of Medicine, University of North Carolina at Chapel Hill, Chapel Hill, NC USA; 13 Collaborative Studies Coordinating Center, Department of Biostatistics, University of North Carolina at Chapel Hill, Chapel Hill, NC USA; 14 Department of Thoracic Medicine and Surgery, Temple University, Philadelphia, PA USA; 15 Department of Medicine, University of Alabama at Birmingham, Birmingham, AL USA; 16 Department of Radiology, University of Iowa, Iowa City, IA USA; 17 Division of Pulmonary and Critical Care Medicine, Johns Hopkins University School of Medicine, Baltimore, MD USA; 18 Department of Internal Medicine, Wake Forest School of Medicine, Atrium Health, Wake Forest Baptist, Winston Salem, NC USA; 19 Department of Medicine, Weill Cornell Medical Center, New York, NY USA; 20 Division of Pulmonary, Critical Care, Sleep and Allergy, University of Illinois at Chicago, Chicago, IL USA; 21 Department of Internal Medicine, Division of Respiratory Medicine, Mayo Clinic, Scottsdale, AZ USA; 22 Division of Respiratory, Critical Care, and Occupational Pulmonary Medicine, University of Utah, Salt Lake City, UT USA; 23 Division of Pulmonary and Critical Care, National Jewish Health, Denver, CO USA; 24 Medical Service, VA Ann Arbor Healthcare System, Ann Arbor, MI USA

**Keywords:** Chronic obstructive pulmonary disease, Systems biology, Proteome informatics

## Abstract

Accelerated progression of chronic obstructive pulmonary disease (COPD) is associated with increased risks of hospitalization and death. Prognostic insights into mechanisms and markers of progression could facilitate development of disease-modifying therapies. Although individual biomarkers exhibit some predictive value, performance is modest and their univariate nature limits network-level insights. To overcome these limitations and gain insights into early pathways associated with rapid progression, we measured 1305 peripheral blood and 48 bronchoalveolar lavage proteins in individuals with COPD [n = 45, mean initial forced expiratory volume in one second (FEV_1_) 75.6 ± 17.4% predicted]. We applied a data-driven analysis pipeline, which enabled identification of protein signatures that predicted individuals at-risk for accelerated lung function decline (FEV_1_ decline ≥ 70 mL/year) ~ 6 years later, with high accuracy. Progression signatures suggested that early dysregulation in elements of the complement cascade is associated with accelerated decline. Our results propose potential biomarkers and early aberrant signaling mechanisms driving rapid progression in COPD.

## Introduction

Chronic obstructive pulmonary disease (COPD), a leading cause of death in the United States^1^, accounts annually for > 600,000 hospitalizations^2^ and $30 billion in direct health expenditures^3^. The course of COPD is heterogeneous. Such heterogeneity is exemplified in part by the highly variable rates of annualized decline in forced expiratory volume in one second (FEV_1_) observed in prospective observational cohort studies^4–7^. This variability between rates of lung function decline causes some individuals to experience relatively stable courses, while others experience accelerated loss of function that leads to severe breathlessness and increased risk of hospitalization and death^6^. Because phenotypic diversity is underpinned by biological heterogeneity, there is growing interest in identifying molecular biomarkers to predict multiple aspects of COPD progression. Such molecular markers could help explain heterogeneity and facilitate the early detection of individuals at risk for accelerated lung function decline, enabling personalized management to arrest disease progression. Biomarkers could also direct research into underlying pathogenic mechanisms to uncover novel therapeutic targets.

To date, accelerated FEV_1_ decline has been associated with individual blood proteins, including club cell secretory protein 16 (CC16)^8,9^, soluble receptor for advanced glycation end-products (sRAGE)^8^, fibrinogen^8^, C-reactive protein (CRP)^8,10^, and interleukin 6 (IL-6)^11^, although contradictory reports exist^10,12–14^. The ratio of leptin to adiponectin in plasma also demonstrated predictive value for rapid lung function decline but showed only moderate sensitivity (63.5%) and specificity (65.1% )^15^. While valuable, univariate analyses of candidate biomarkers have provided limited insights into underlying mechanisms of progression, a shortcoming that could be complemented by network-level analysis of large numbers of proteins.

Previous studies have also been limited to measuring biomarkers in a single tissue compartment due to sample availability. However, data indicate that combinations of proteins appear to be better predictors of multiple COPD outcomes (including FEV_1_ decline) than individual factors^8^, especially when derived from multiple compartments. Cross-sectional studies integrating datasets across multiple molecular levels and anatomical locations has improved the ability to classify individuals with a smoking history by COPD status and uncovered novel disease-associated pathways^16–18^. Accordingly, analyses of lung function decline may benefit from evaluating systems-level integrated networks that are more likely to capture the diverse biology driving airflow obstruction^19,20^.

Data-driven modeling is one approach that will enable inference of network-level relationships driving progression. By permitting data integration across multiple tissue compartments, data-driven modeling generates systemic networks (“signatures”) of co-varying biological factors associated with disease phenotypes. Identified signatures can be linked to pathogenic mechanisms, providing insight into potential targets for follow-up experiments or as biomarkers for therapeutic intervention. These approaches have been successfully used to identify blood and bronchoalveolar lavage (BAL) protein signatures associated with disease state and progression in idiopathic pulmonary fibrosis^21,22^. We have also used them to integrate blood and sputum protein signatures associated with COPD exacerbation^23^.

Here, to gain insights into cross-compartment mechanisms associated with a greater lung function decline in COPD, we applied an integrative data-driven modeling pipeline to proteins recovered from matched blood and BAL samples from participants in the bronchoscopy sub-study^24,25^ of the SubPopulations and InteRmediate Outcome Measures In COPD Study (SPIROMICS)^26^. Our results suggest that proteomic signatures can effectively detect individuals at increased risk of accelerated lung function decline. They also provide insights into COPD progression mechanisms that can be further investigated in validation cohorts and follow-up murine studies.

## Results

### Participant characteristics

**Figure 1:**
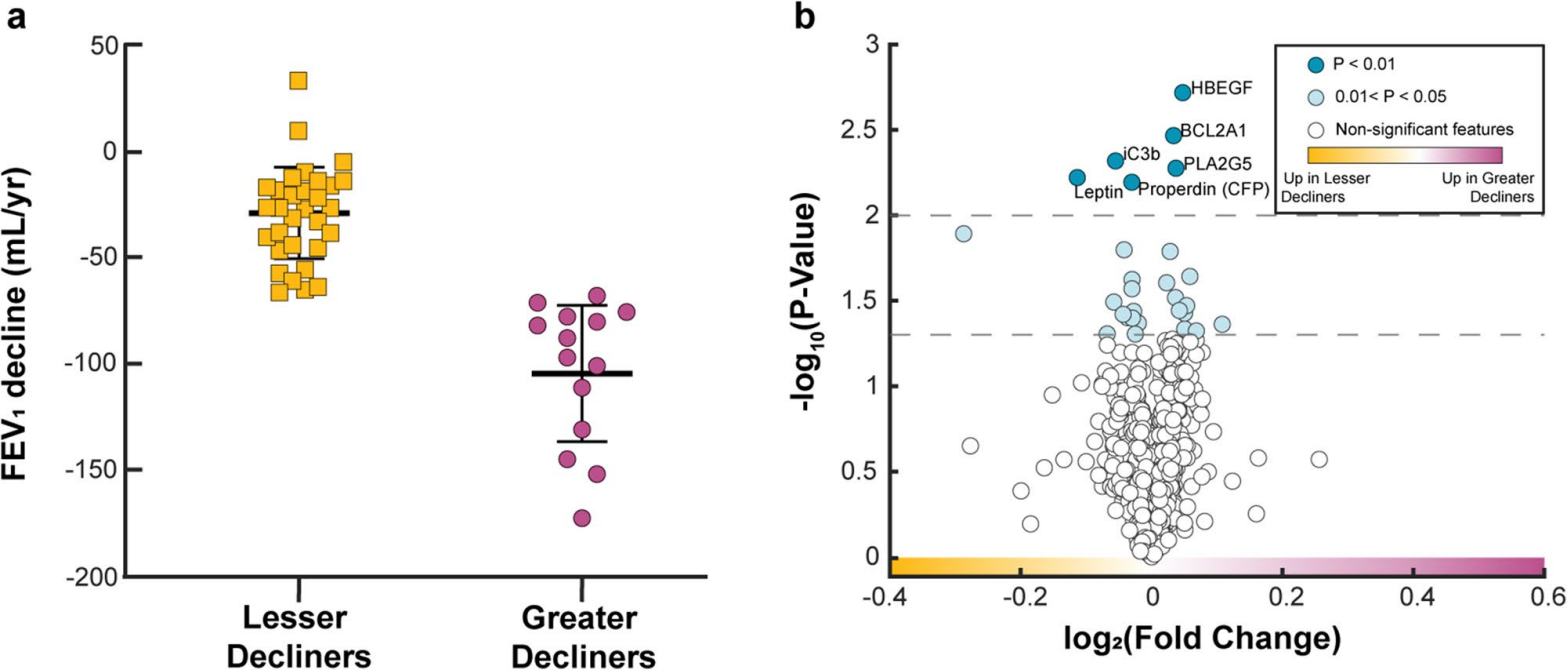
Individual blood and BAL proteins cannot discriminate between annualized greater versus lesser rates of FEV_1_ decline in COPD. (**a**) Comparison of annualized post-bronchodilator FEV_1_ decline from V1 to V5. Decline was calculated as (V5 FEV_1_ – V1 FEV_1_)/ time, where time is the duration in years between V1 and V5 for each participant. (**b**) Volcano plot of blood and BAL proteins. Light and dark blue protein markers have a p-value < 0.05 and < 0.01, respectively, after a two-sampled two-tailed t-test. All depicted p-values are before correction for multiple comparisons. No proteins remained significant after applying the Benjamini-Hochberg false discovery rate (FDR) correction for multiple comparisons (α = 0.05).

We evaluated participants of the SPIROMICS bronchoscopy sub-study who had COPD, paired baseline (Visit 1/ V1) and final (Visit 5/ V5) spirometry, plus matched proteomic measurements from plasma samples and BAL samples (n = 45) (Supplementary Fig. S1). Participants’ mean (± SD) age at V1 was 63 ± 7.7 years; they had a follow-up time of 6.3 ± 0.9 years (Table 1). To characterize rapid progression, we dichotomized participants based on their annualized FEV_1_ decline (∆FEV_1_): greater decliners (< 30th percentile) (n = 14) versus lesser decliners (≥ 30th percentile) (n = 31) (Fig. 1a). This threshold equaled a ∆FEV_1_ of –70 mL/year. Greater decliners trended non-significantly to be male (85.7% vs. 54.8%) but were well-matched for other demographic criteria. Despite significantly higher FEV_1_% predicted (*p* = 0.017) and absolute FEV_1_ (*p* = 0.015) at V1, they experienced a 3.6-fold greater ∆FEV_1_ compared to their lesser decliner counterparts (− 104.6 ± 32.0 vs. − 28.8 ± 21.5 mL/year). The observed association between faster decline and higher baseline lung function is in line with previous reports^4,27^.

**Table 1:** Baseline characteristics of COPD cases.

		All (N = 45)	Greater decliners^‡^ (N = 14)	Lesser decliners (N = 31)	P-Value^†^
Age*		63.4 (± 7.75)	64.2 (± 6.24)	63.1 (± 8.41)	0.65
Currently Smoking*		15 (33.3%)	5 (35.7%)	10 (32.3%)	> 0.99
BMI*		27.8 (± 4.91)	27.9 (± 3.67)	27.8 (± 5.43)	0.93
Sex (Male)		29 (64.4%)	12 (85.7%)	17 (54.8%)	0.09
Race (White / Other)		37/8 (82.2%)	12/2 (85.7%)	25/6 (80.6%)	> 0.99
ICS use* (yes)		17 (37.8%)	3 (21.4%)	14 (45.2%)	0.18
FEV_1_* (% predicted)		75.6 (± 17.4)	**84.2** **(± 13.1)**	**71.1** **(± 17.7)**	**0.017**
FEV_1_/FVC*		0.58 (± 0.09)	0.60 (± 0.08)	0.57(± 0.10)	0.27
FEV_1_* (L)		2.27 (± 0.68)	**2.63** **(± 0.60)**	**2.11** **(± 0.66)**	**0.015**
Visit 5 FEV_1_ (L)		1.94 (± 0.67)	1.97 (± 0.67)	1.93 (± 0.68)	0.85
Time from baseline to Visit 5 (yrs)		6.31 (± 0.86)	6.25 (± 0.76)	6.33 (± 0.91)	0.79
Time from baseline to bronchoscopy (months)		20.3 (± 11.6)	20.4 (± 10.0)	20.3 (± 12.5)	0.96
∆FEV_1_ (mL/yr)		− 52.4 (± 43.3)	**− 104.6** **(± 32.0)**	**− 28.8** **(± 21.5)**	

Two-sample, two-tailed t-test or Fisher’s exact test were used to determine significant differences.

*Demographic information from baseline visit (Visit 1).

^†^P-values are associated with differences between greater decliner and lesser decliner groups.

^‡^Decline in FEV_1_ (mL/yr) ≥ 70 mL/year (see Methods).

### Individual blood and BAL proteins cannot discriminate between rates of longitudinal lung function decline

We first determined whether differences existed between greater decliners and lesser decliners in multi-compartment protein expression measured early in the study, using the concentrations of 1305 blood and 25 BAL proteins measured with SOMAScan and Luminex technology, respectively. To reduce biases associated with the unequal distribution of women across classes (14.3% vs. 45.2%), we first removed proteins (n = 8) that exhibited significant associations with sex (Supplementary Fig. S2). Across the remaining 1322 proteins, 28 (2.1%) had a mean concentration that differed significantly (*p* < 0.05) between groups (Fig. 1b). Of these, 13 were increased in greater decliners [log_2_ fold change (FC) > 0] and 15 were increased in lesser decliners (FC < 0). The top six most significantly different proteins (*p* < 0.01) were all identified in blood: Heparin-binding EGF-like growth factor (HBEGF; FC = 0.05), BCL2-related protein A1 (BCL2A1; FC = 0.03), inactivated Complement C3b (iC3b; FC =  − 0.05), Calcium-Dependent Phospholipase A2 (PLA2G5; FC = 0.03), Leptin (FC =  − 0.11), and Properdin (CFP; FC =  − 0.03). None of the 28 proteins remained significant after Benjamini–Hochberg adjustment.

### Data-driven modeling identifies a proteomic multi-compartment signature that prospectively differentiates progression phenotypes

We next used data-driven modeling approaches to identify a multi-compartment signature of co-varying proteins associated with greater FEV_1_ decline. Here, we combined all 1322 protein measurements (1297 blood and 25 BAL) into a single dataset, then applied elastic net (EN) in tandem with partial-least squares discriminant analysis (PLSDA). First, EN regularization was applied iteratively to 2000 subsets of randomly resampled data. Then, based on their selection frequency throughout the iterations, proteins were ranked (most to least frequent) and fed stepwise into the PLSDA algorithm. We evaluated PLSDA model performance at each step using sixfold cross-validation (CV) and selected the model with the highest CV accuracy as the optimal signature.

**Figure 2:**
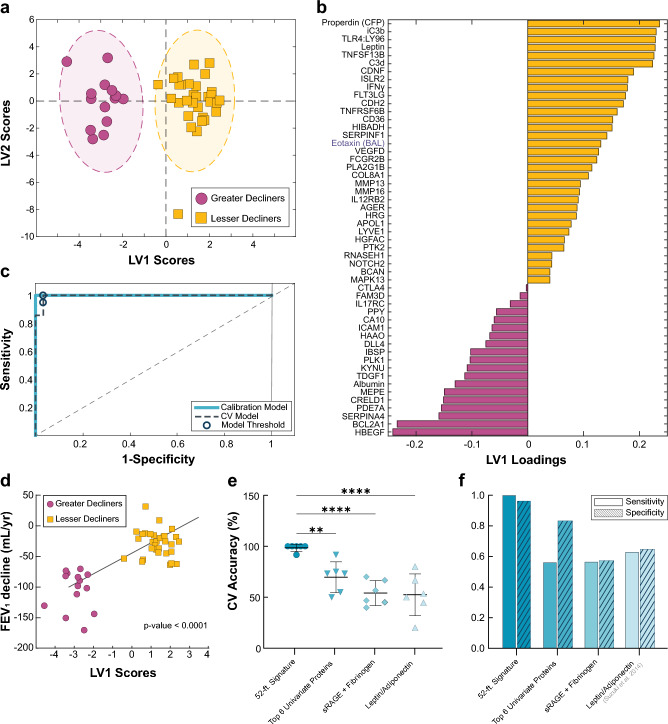
A 52-feature Elastic Net (EN) signature identified individuals at-risk for FEV_1_ decline ≥70 mL/year with high accuracy. (**a**) PLSDA scores plot highlighting strong differentiation between greater decliners (magenta) and lesser decliners (yellow), separating the two groups with 98.4% cross-validation (CV) and calibration accuracy. (**b**) Loadings on latent variable 1 (LV1) (with negatively loaded proteins being comparatively increased in greater decliners and positively loaded proteins being comparatively reduced) captured 11.9% of the total variance in the data. (**c**) ROC curve of 52-feature signature suggests greater decliners classification with 100% sensitivity and 96.8% specificity in the cross-validated model. (**d**) LV1 scores were associated with annualized FEV_1_ decline (mL/yr). P-values and fit line shown for linear models adjusted for age, race, height, sex, baseline FEV_1_% predicted, smoking status, pack-years, and inhaled corticosteroids (ICS) use within three months of baseline visit. (**e**,**f**) Comparison of (**e**) 6-fold CV accuracies, (**f**) sensitivities, and specificities between the 52-feature EN signature, a collection of the six top proteins identified in Fig. 1, and literature-based models. All reported values are from cross-validated PLSDA models, unless otherwise noted. One-way ANOVA with Dunnett’s post hoc test; **p<0.01, ****p < 0.0001.

Using this feature selection pipeline, we identified a signature of 52 proteins (51 blood and 1 BAL) that distinguished greater decliners (FEV_1_ decline ≥ 70 mL/year), along the latent variable 1 (LV1) axis, with 98.4% calibration and CV accuracy, 100% sensitivity, and 96.8% specificity (Fig. 2a-c). Permutation tests performed on participant scores across the first two principal components of PCA models generated with the 52-feature signature, show no significant influence of baseline ICS use (*p* = 0.35) or smoking status (*p* = 0.57) on participant classification (Supplementary Fig. S3). To confirm the accuracy of the selected features, we compared its cross-validated accuracy to 1000 random signatures, generated by selecting iterative groups of 52 random proteins from the original dataset. None of the random signatures outperformed the optimal model (*p* < 0.0001) (Supplementary Fig. S4). We also observed a significant, albeit moderate, Pearson correlation between LV1 scores and plasma concentrations of CRP (r_p_ = 0.33, *p* = 0.03), a protein previously associated with FEV_1_ decline^8,10^. However, LV1 scores exhibited no significant correlations with other reported blood markers of spirometric decline, including IL-6^11^ (r_p_ = 0.18, *p* = 0.29), fibrinogen^8^ (r_p_ =  − 0.01, *p* = 0.93), and matrix metalloproteinase 9 (MMP-9)^10^ (r_p_ =  − 0.27, *p* = 0.08).

Lack of a formal definition of “rapid progression” in COPD has led to literature variability, so we explored whether the identified 52-feature signature maintained significance across alternative characterization approaches. Recently, using the entire SPIROMICS dataset, Anderson et al. proposed a threshold-based definition, classifying progression into three groups based on annualized FEV_1_ declines: rapid decliners (> 100 mL/year), decliners (20–100 mL/year), and stable/ improvers (< 20 mL/year)^28^. The limited number of subjects in our bronchoscopy sub-study exhibiting such extreme decline hindered direct exploration of this definition. However, an exploratory PCA using the 52-feature multi-compartment signature demonstrated significant signature enrichment in rapid decliners thus defined (Supplementary Fig. S5), suggesting our model reliably extends to this more rigorous definition.

A regression-based analysis also found that participant scores on LV1 correlated highly with annualized declines in FEV_1_ (r_p_ = 0.758, *p* < 0.0001), even after adjustment for age, race, height, sex, baseline FEV_1_% predicted, smoking status, pack-years, and ICS use (*p* < 0.0001) (Fig. 2d). Alternative estimations of FEV_1_ decline using all available longitudinal spirometry (rather than just V1 and V5) produced similar results, as did classifications using FEV_1_% predicted in lieu of absolute FEV_1_ volumes (Supplementary Fig. S6). These results suggest that our approach captures complex progression trends and is largely not skewed by demographic factors influencing lung capacity. For the remainder of our analysis, we use the -70 mL/year definition.

To contextualize the performance of the 52-feature multi-compartment signature, we compared it to cross-validated analyses based on proteins identified in the univariate analysis (Fig. 1b) and the literature. Unsurprisingly, the 52-feature signature performed significantly better than independent PLSDA models generated with each of the top six most differentially expressed proteins (Supplementary Fig. S7) and a combinatorial model generated using all six (Fig. 2e). The signature also significantly outperformed cross-validated models built with published combinations of proteins associated with spirometric decline (sensitivity, specificity: sRAGE and Fibrinogen, 57.1%, 58.0%; Leptin: Adiponectin ratio, 63.5%, 65.1%) (Fig. 2f). To explore how the identified signature characterize controls with relation to COPD subjects, we generated an additional PCA with the 52 signature-identified proteins and a reference groups of tobacco-exposed people with preserved spirometry (TEPPS) (n = 38) who have a history of smoking but no airflow obstruction (FEV_1_/FVC > 0.7) (Supplementary Table S1). Using a permutation test with participants' scores across the first two principal components, we show that TEPPS responses were significantly different from Greater Decliners (*p* < 0.0001) but not from Lesser Decliners, suggesting our signature can uniquely characterize smoking controls from Greater Decliners alone (Supplementary Fig. S8).

### The progression signature is enriched for proteins involved in the complement system

**Figure 3:**
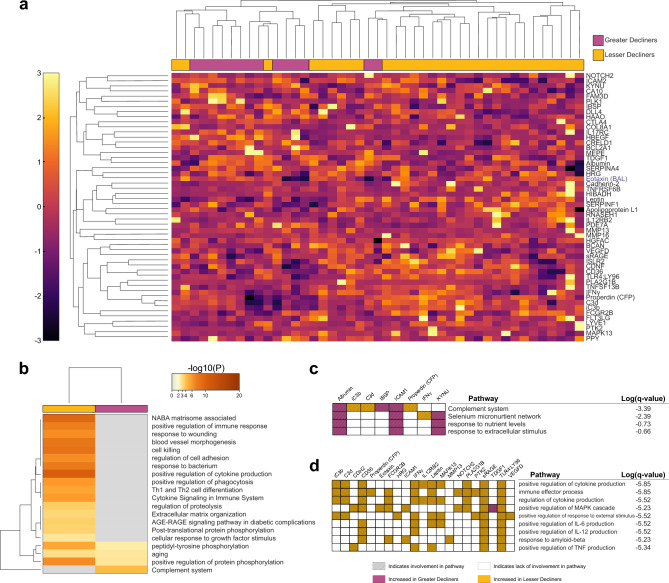
Clustering of COPD subjects by the EN-identified signature highlights distinct regulation of immune-associated processes. (**a**) Hierarchical clustering of the 52-feature signature highlights distinct clustering of greater decliners (magenta) and lesser decliners (yellow). Only 5 out of the 45 subjects were misclassified (Sensitivity: 85.7%, Specificity: 90.3%). BAL proteins denoted by blue text. (**b**) Significantly enriched ontology clusters by Metascape analysis. (**c**, **d**) Pathways encompassed in the (**c**) complement system cluster and in the (**d**) positive regulation of cytokine production cluster are listed in the table. Hatched squares indicate protein involvement in a particular pathway, colorations of magenta or yellow represent a relative elevation of the protein concentration in greater decliners or lesser decliners, respectively.

Having generated a high-performing progression signature, we sought to understand the biological implications of its components. Unsupervised hierarchical clustering identified greater decliners with 88% accuracy (Fig. 3a). A Metascape analysis found 20 significantly enriched ontology clusters (Fig. 3b), with only three, related to aging and phosphorylation-dependent signal transduction, shared between groups. Lesser decliners displayed unique enrichment of 16 clusters related primarily to inflammation and immune functions. Notably, the only uniquely enriched cluster in greater decliners was associated with the complement system (q = 4.10e-04) (Fig. 3c-d). Proteins in this cluster included blood albumin, bone sialoprotein 2 (IBSP), intracellular adhesion molecule 1 (ICAM1), interferon-gamma (IFN-γ), kynureninase (KYNU), and three complement proteins (iC3b, C3d, Properdin). These three complement proteins were involved in 10 of 20 total clusters and represented three of the top eight loaded proteins in the PLSDA, indicating a potentially significant impact of complement processes in COPD progression.

### Dysregulated complement protein signatures are associated with accelerated FEV_1_ decline

**Figure 4:**
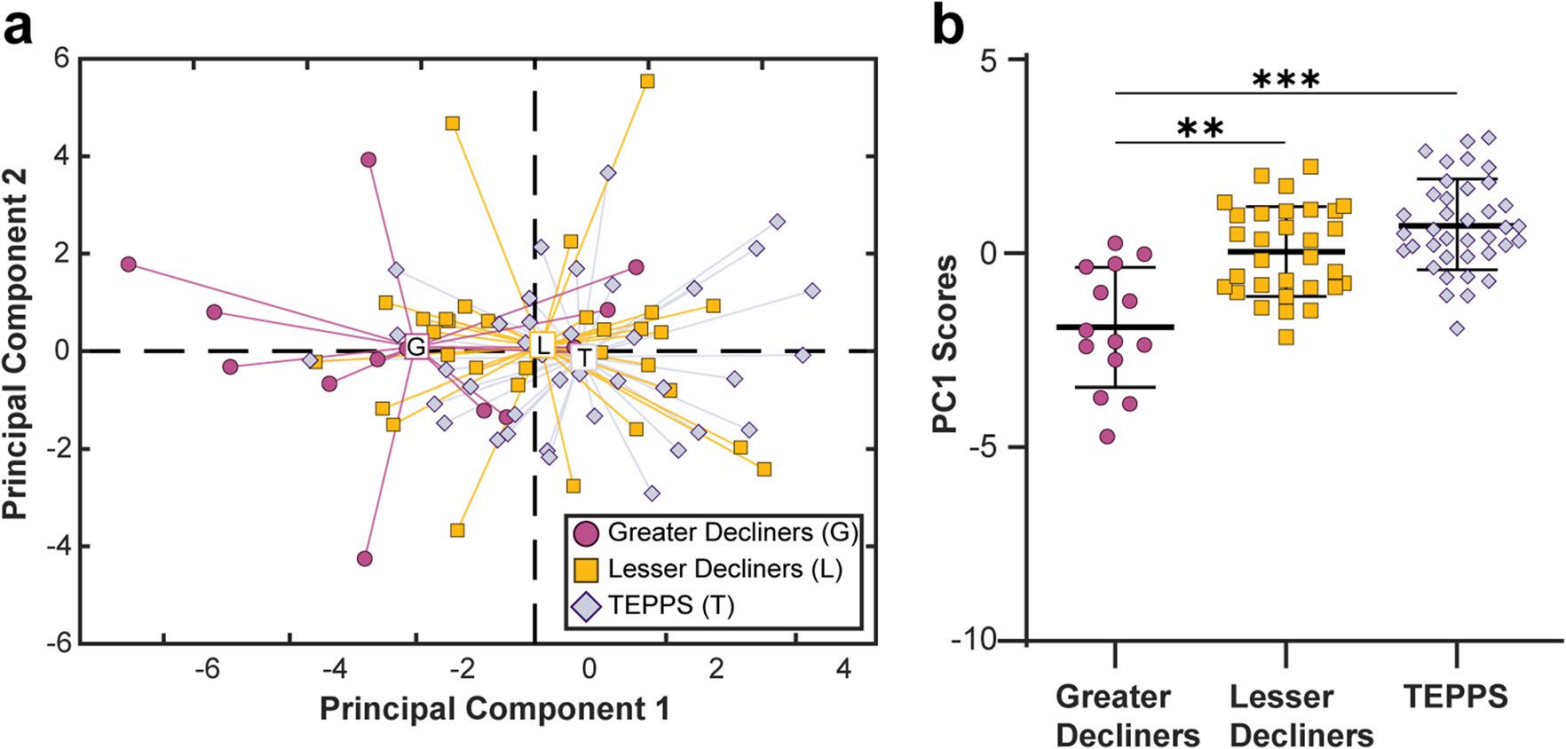
Complement profiles in COPD lesser decliners behave more similarly to TEPPS than COPD greater decliners. (**a**) Scores plot from PCA completed using all complement proteins measured in plasma (C1q, C1qBP, C1r, C2, C3d, C3b, C3, C3a, iC3b, C3a des Arg, C4, C4b, C5, C5a, C5-6, C6, C7, C8, C9, Factor B, Factor D, Properdin) of greater decliners (circles), lesser decliners (squares), and a reference group of tobacco-exposed people with preserved spirometry (TEPPS) (diamonds). First two principal components (PCs) capture 33.9% of the variance in the dataset. (**b**) Comparison of scores on PC1 (one-way ANOVA with Tukey’s post-hoc test; **p<0.01, ***p<0.001).

These enrichment data, coupled with the known importance of the complement cascade to immunity, suggest that complement proteins may be more globally altered in greater decliners than was captured in the original signature. To explore this possibility and focus on complement dysregulation, we performed PCA using the concentration of 22 complement proteins measured in plasma. Results suggested that progression groups differed significantly in complement profiles as measured by scores across PC1 (*p* = 0.0045) (Supplementary Fig. S9). We selected PC1 scores as the PC of interest, as only they correlated significantly with annualized FEV_1_ decline (*p* < 0.001) (Supplementary Fig. S10a). To explore whether the observed patterns were specific to the greater decliners rather than COPD more generally, we extended our analysis to include the reference group of TEPPS (n = 38). Interestingly, the baseline profiles of TEPPS were similar to that of lesser decliners. In contrast, complement profiles from greater decliners differed from those of lesser decliners and TEPPS, both as visualized using PCA (Fig. 4a) and via direct comparison of PC1 scores (Fig. 4b). The variance in complement profiles, as captured by PC1, correlated with FEV_1_ decline (*p* = 0.004) (Supplementary Fig. S10b), a relationship that remained significant after adjustment for age, race, height, sex, FEV_1_% predicted, smoking status, pack-years, and ICS use (*p* = 0.012) (Supplementary Fig. S10c). In univariate comparisons, among the 22 complement proteins, only C1r, iC3b, C3d, Properdin, and C4 reached statistical significance (Supplementary Fig. S11). Performing permutation tests with participant scores across the first two principal components labeled by key clinical variables, we show no significant influence of baseline ICS use (*p* = 0.36) or current smoking status (*p* = 0. 70) on observed complement profiles (Supplementary Fig. S12). Collectively, these findings reinforce the Metascape results, suggesting that early patterns of complement dysregulation are specific to a more rapidly progressing phenotype.

### Alternative minimal signatures highlight a small number of proteins that maintain high predictive power

**Figure 5:**
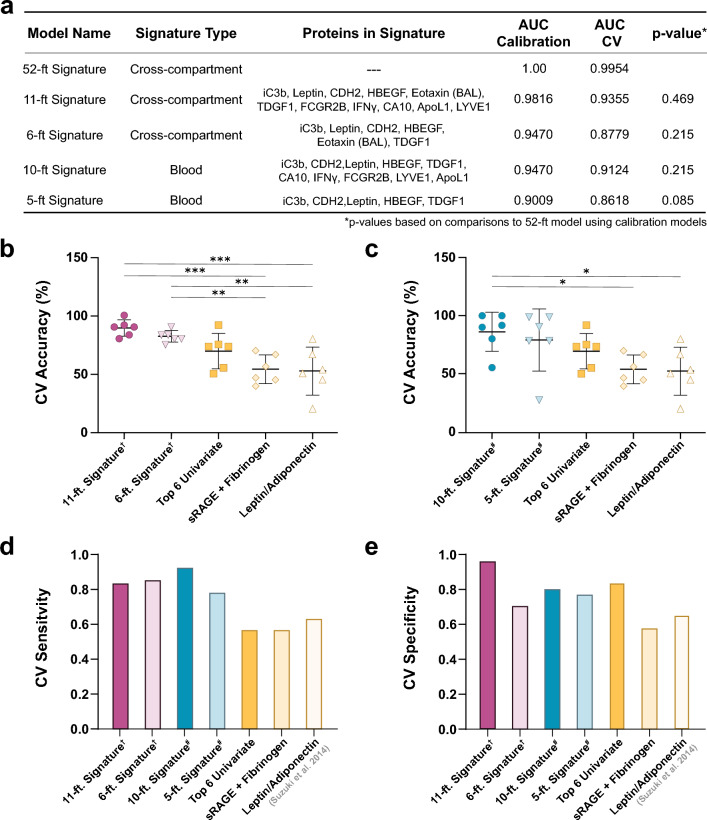
Select subgroup of signature proteins retain high predictive value for accelerated FEV_1_ decline. (**a**) Table denote AUCs for both ROC curves generated from calibration and cross-validation PLSDA models. AUCs of all calibration models were compared to that of the optimal (52-feature model) using the Hanley and McNeil method^58^. (**b**) Comparisons of 6-fold CV accuracies of multi-compartment and (**c**) blood-only biomarker models to a collection of the 6 top proteins identified in Fig. 1, and literature models, as determined by ANOVA with Bonferroni’s post hoc test (*p<0.05, **p < 0.01, ***p<0.001). (**d**) Sensitivity and (**e**) specificity of signatures. All reported values are from cross-validated PLSDA models, unless otherwise noted. (†: multi-compartment model, #: blood-only model). CV: cross-validated; AUC: area under curve.

Although the large number of proteins in the differentiating signature proved advantageous in exploring functional enrichments associated with accelerated FEV_1_ decline, it is too large to be used as a prediction tool. Therefore, we next explored whether smaller sub-models (“minimal signatures”) maintained predictive value. Using insights from our step forward PLSDA pipeline, we visually analyzed the trade-off between model size and performance. Two minimal signatures, with 6 and 11 features, respectively, had CV accuracies similar to our optimized model (CV accuracy: 6-feature, 81.6%; 11-feature, 88.4%; 52-feature, 98.4%) (Supplementary Fig. S13a). The 6-feature model consisted of BAL Eotaxin and blood iC3b, Leptin, Cadherin-2, HBEGF, and TDGF1. The 11-feature model comprised those six, plus blood-derived FCGR2B, IFN-γ, CA10, Apolipoprotein L1, and LYVE1. ROC curves created for the 11- and 6-feature models resulted in areas under the curve (AUC) of roughly 0.982 and 0.947 for the calibration models and 0.935 and 0.878 for the cross-validated models, respectively (Fig. 5a; Supplementary Fig. S14). Intriguingly, each minimal signature included at least one protein from both plasma and BAL.

We tested proteins from both blood and alveolar compartments to maximize the chances of understanding underlying biology in COPD, specifically the link between distal airways and systemic events. However, due to the invasiveness of bronchoscopy, biomarkers obtained solely from the blood would be preferable. Accordingly, we evaluated a minimal blood signature by applying our step forward EN/PLSDA algorithm exclusively to the 1297 blood proteins measured in the same COPD participants (n = 45). Analysis identified 5- and 10-feature signatures that maintained strong cross-validated performance (CV accuracy: 5-feature, 81.6%; 10-feature, 86.8%) (Supplementary Fig. S13b). The 5-feature model included blood-derived iC3b, Cadherin-2, Leptin, HBEGF, and TDGF1; the 10-feature model comprised those five, plus CA10, IFN-γ, FCGR2B, LYVE1, and Apolipoprotein L1. Both minimal blood signatures displayed a slight drop in performance compared to their multi-compartment counterparts of nearest sizes (10- and 5-feature signatures, AUC 0.947, 0.901 for calibration models and 0.912, 0.862 for CV models, respectively). However, comparisons using both AUCs and sixfold cross-validation found no significant difference in the performance of any of the minimal signatures and the optimal 52-feature model (Fig. 5a; Supplementary Fig. S15).

Finally, we subjected the minimal signatures to cross-validated analyses relative to multivariate signatures identified through our univariate analysis or published literature. Comparisons using sixfold CV accuracy indicated that overall performance was largely sustained. The 6- and 11-feature multi-compartment signatures and the 10-feature blood signature significantly outperformed literature-based models and trended towards outperforming a signature based on the top 6 univariate proteins (Fig. 5b-c). The 5-feature blood signature did not reach statistical significance in any comparison, though its performance over literature-based models was substantially improved. All four signatures showed a > 20% increase in sensitivity, with lesser though considerably improved specificity versus other models (Fig. 5d-e). Collectively, these findings imply that small, proteomic signatures can differentiate individuals with tobacco smoking-associated COPD at risk for rapid lung function decline with high accuracy.

## Discussion

This study used two complementary datasets from COPD participants in the SPIROMICS bronchoscopy sub-study to generate insights into early, aberrant signaling mechanisms associated with accelerated lung function decline. Using a systems analysis, we identified a multivariate signature of early blood and BAL proteins that predicted individuals at-risk for FEV_1_ declines ≥ 70 mL/year (“greater decliners”) with > 98% accuracy. Investigation of this signature disclosed that differences in longitudinal FEV_1_ decline are associated with variability in host immune and defense responses, with greater decliners uniquely exhibiting early dysregulated patterns of complement protein expression. Finally, refinement of the signature identified a minimal model with 10 blood proteins that, if validated, may serve as a clinically feasible prognostic tool. This work complements previous predictions of COPD progression^8–14^ by starting from a data-driven approach, rather than prior knowledge, to obtain unbiased insights into cross-compartment proteins and pathways driving accelerated airflow obstruction.

To our knowledge, this is the first longitudinal study of COPD progression to use integrated proteomic datasets derived from blood and BAL samples. Our tandem EN and PLSDA approach identified concise proteomic signatures from thousands of proteins measured across multiple tissue compartments. This framework accurately differentiated individuals with COPD who sustained declines in lung function ≥ 70 mL/year based on proteins measured early after participant enrollment. We specifically sought to explore integrated lung and systemic compartment models because COPD has coupled local and peripheral manifestations^29^. Although several proteins were measured in lung and blood tissue compartments, classifying signatures did not select any matched BAL/blood proteins, which may be partly due to differences in measurement platforms. Moreover, the fact that only one BAL protein was identified in the 52-feature signature is likely in part a consequence of the marked difference in numbers of analytes in blood and BAL (1297 vs. 25). Currently, the SOMAmer technology used in our plasma analyses has not been validated for use in BAL samples. However, the importance of multi-compartment representation is exemplified by the over 15% improvement in calibration model sensitivity on adding BAL Eotaxin to the 5-feature blood signature.

Our multi-compartment 52-protein signature was enriched for inflammation and immune responses processes, consistent with the central role of immune dysregulation in COPD pathogenesis^30–32^. Chief among these processes was the complement system. By providing evidence that complement alterations precede accelerated FEV_1_ decline, we extend previous associations between the levels of blood-derived complement proteins (C3^33,34^, C4^35^, C4b^36^, C5a^37^, C9^38^, Factor B^36^) and COPD status (case vs. control), cross-sectional analyses of FEV_1_% predicted^39,40^, and emphysema severity^41^. How such aberrations might contribute to airflow limitation is unknown. In a murine model, C3 cleavage contributed to smoking-induced emphysema via an influx of conventional dendritic cells^42^, a cell type that can initiate both innate and adaptive immune responses. Our findings regarding involvement of complement proteins are specific to blood. Although our chosen assay system could not analyze complement components in BAL, complement dysregulation might extend into the lung, as suggested by altered levels of C5a in the sputum in COPD^37,43^ and airway C3 deposits in lungs in smoking-associated emphysema^42^. Airway epithelial cells have also been shown to secrete and store C3, suggesting the presence of a localized C3 supply that may aid host defense^44^. The observed global patterns of complement dysregulation were robustly associated with FEV_1_ decline, but SomaLogic aptamers cannot reliably distinguish between complement cleavage products and their parent proteins. Hence, we cannot explore the relationship between global complement levels and pathway activation. However, our observation of complement dysregulation before FEV_1_ decline provides compelling temporal evidence that the complement pathway contributes to accelerated progression.

Focusing on eventual clinical feasibility, we identified an alternative parsimonious 10-protein signature derived using only peripheral blood; its potential prognostic value, if validated, is suggested by its superior performance to reported multivariate biomarkers of FEV_1_ decline^8,15^. To our knowledge, except for leptin^15^, these proteins have not been associated with accelerated loss of lung function. Consistent with previous studies^8–11^, the proteins in this signature are primarily related to immune and inflammation responses (leptin, iC3b, IFN-γ, FCGR2B, APOL1). However, we also observed notable contributions from endothelial-mesenchymal transition (EMT) proteins (Cadherin-2 and HBEGF). EMT is active in both large and small airways of COPD and relates to airflow obstruction^45–47^. Cadherin-2 is increased in epithelial cells from COPD subjects as compared to healthy controls^48^. Similarly, serum and sputum HBEGF levels have been positively associated with COPD severity measures, including FEV_1_% predicited^49^ and CAT score^50^. The final proteins involved in the signature (TDGF1, LYVE1, CA10) have not previously been associated with COPD. However, in lung cancer, which is thought to share overlapping etiologic features, TDGF1 (increased in greater decliners) predicts poor progression-free survival^51^, while LYVE1 (increased in lesser decliners) is associated with reduced metastasis and mortality^52^. Even in this parsimonious signature, we observed diverse biological enrichment. These findings emphasize the value of multivariate signatures in evaluating heterogeneous conditions such as COPD, where abnormalities in several pathways or pathway constituents likely drive a singular clinical outcome.

Our study has limitations. Chief among these is the lack of a validation cohort as, to our knowledge, none currently exists with both BAL protein measurements and longitudinal follow-up. Missing data also limited our sample size. BAL was not collected successfully on all participants, chiefly due to airway collapse during the procedures, and not all participants completed V5. Hence, we have relatively small numbers of participants, disproportionately non-Hispanic whites. To mitigate the influence of individual participants, we applied an iterative bootstrapping framework during model generation. Still, the generalizability of our findings remains unclear, given the issues of limited heterogeneity based on sample size and demographics. We recognize that we cannot definitively conclude whether the identified signatures precede lung function decline, as decline may have been ongoing prior to baseline sample acquisition. However, the modestly higher baseline FEV_1_ observed in greater decliners support this possibility. Additionally, the cross-sectional nature of our proteomic data limits any insight into the temporal stability of our identified signatures. Because there is no universally agreed-upon definition of rapid progression, we used a percentile cut-off in FEV_1_ decline, as used by others^15,53^; resulting in a cut-off (≥ 70 mL/year) similar to previous reports. However, other threshold-based definitions (i.e., FEV_1_ decline > 100 mL/year) have been proposed^28^. Few participants (n = 6) experienced declines > 100 mL/year in our data, providing insufficient power to investigate this definition accurately. However, in an exploratory PCA, we show that our 52-feature multi-compartment is enriched significantly in participants with declines > 100 mL/year, suggesting our signature extends to this more stringent characterization. Moreover, while we acknowledge that a fixed cut-off definition may favor an overrepresentation of males as greater decliners due to physiologic differences in lung function measurements between sexes, we are underpowered to explore sex-stratified analyses. Still, alternative estimations of FEV_1_ decline using FEV_1_% predicted, which accounts for age, sex, and body composition, in lieu of absolute FEV_1_ volumes, produced similar results, suggesting a minimal impact of sex on group affiliation in this study. Lastly, a longitudinal decline in FEV_1_ is only one parameter that can evaluate progression and is less sensitive to capturing changes in small airway loss than other clinical measures, like parametric response mapping. However, it is worth noting that elements of our signature may reflect potential markers of small airway damage, as our signature exhibits enrichment of several processes that contribute to small airway damage, such as the response to wounding and ECM organization^54^. Nonetheless, future studies are needed to explore the relevance of the identified signature in assessing other outcomes.

In summary, data-driven modeling approaches identified early cross-tissue compartment proteomic signatures and provided insight into potential mechanisms associated with accelerated disease progression in COPD. This work highlights the ability of quantitative, systems-focused analytical techniques to accomplish both these goals. Data-driven modeling approaches could be applied to integrate spatiotemporal data in clinical samples from other diseases with a progressive or heterogeneous population.

## Methods

### Human participants

SPIROMICS (ClinicalTrials.gov Identifier: NCT01969344) is an ongoing multicenter, prospective observational study designed to identify new COPD subgroups and intermediate biomarkers of disease progression^26^. Briefly, we enrolled participants aged 40–80 years at entry with a history of cigarette smoking (≥ 20 pack-years), either with COPD by the fixed ratio definition (post-bronchodilator FEV_1_/FVC < 0.7), or without COPD; as controls, we recruited healthy individuals without smoking history. SPIROMICS participants (n = 2,974) underwent a baseline examination (V1) followed by yearly visits for up to three years and a final follow-up visit (V5) approximately 5–8 years after V1. The first participant entered on November 10, 2010, and we censored all data on July 31, 2021. The study was conducted according to the principles of the Declaration of Helsinki. The human study protocol was approved by the institutional review board of all participating centers and methods were carried out in accordance with the relevant guidelines and regulations (Columbia University, New York, NY, United States; Johns Hopkins University, Baltimore, MD, United States; National Jewish Health, Denver, CO, United States; Temple University, Philadelphia, PA, United States; University of Alabama at Birmingham, Birmingham, AL, United States; University of California Los Angeles, Los Angeles, CA, United States; University of California San Francisco, San Fransico, CA, United States; University of Illinois at Chicago, Chicago, IL, United States; University of Iowa, Iowa City, IA, United States; University of Michigan, Ann Arbor, MI, United States; University of North Carolina at Chapel Hill, Chapel Hill, NC, United States; University of Utah, Salt Lake City, UT, United States; Wake Forest University, Winston Salem, NC, United States). All participants were aware of the study’s intent and provided written informed consent before any procedures.

Some SPIROMICS subjects (n = 215) from all groups except those with severe (GOLD 4) COPD participated in a bronchoscopic sub-study^24,25^, which included BAL of the right middle lobe and lingula. Participants (n = 149) with a history of smoking who had available blood and BAL samples were considered for inclusion in the initial Elastic Net analysis (Supplementary Fig. S1). This analysis was restricted to participants (n = 85) of that sub-study who had a history of smoking, available spirometry that did not improve at V5, and full biospecimens, which were plasma samples at V1 and BAL cytokine analysis; their baseline characteristics are shown in Table 1. They comprised two study groups: COPD cases (n = 45) and a reference group (n = 40) of TEPPS who had no airflow obstruction at both V1 and V5 (Supplementary Table S1). The demographics of our study participants (n = 85) did not differ significantly from the entire SPIROMICS bronchoscopy cohort (Supplementary Table S2).

Based on the magnitude of annual change in FEV_1_, we dichotomized the COPD cases into greater decliners (< 30th percentile; n = 14) versus lesser decliners (≥ 30th percentile; n = 31). FEV_1_ decline was calculated using the two-point slope equation: [*V5 FEV*_*1*_*—V1 FEV*_*1*_]/*time*, where *time* is the duration, in years, from V1 to V5 for each subject. Time calculations assumed a fixed-length year equal to 365.2425 days.

### Sample preparation & datasets

*Blood dataset*: Fresh plasma samples collected at V1 were frozen in either an EDTA collection tube or a P100 tube with K2EDTA^55,56^. SOMAmer© (slow off-rate modified aptamer) technology^57^ (SomaLogic, Boulder, CO) was used to measure 1305 proteins from participants in the SPIROMICS bronchoscopy sub-study.

*BAL dataset*: We measured the concentration of 48 proteins including cytokines, chemokines, and growth factors (HCYTA-60 K-PX48, Milliplex, EMD Millipore Corporation) in BAL aliquots from a subset of participants in the SPIROMICS bronchoscopy sub-study (n = 184) using Luminex FlexMAP 3D (Luminex Corporation, Austin, TX) technology. Any results above the upper limit of detection were set to the maximum detectable concentration of that analyte. We set samples below the lower limit of detection to be half the lowest minimum detectable concentration across the standard curves of all analytes. We removed the 23 proteins in which ≥ 50% of measurements were below the lower limit of detection across all samples, yielding 25 analyzable BAL proteins. Before analysis, we normalized all BAL protein concentrations to the total BAL protein concentration of the respective sample, as quantified by a Pierce BCA Protein Assay Kit (Pierce Protein Biology, Rockford, IL).

*Multi-compartment dataset*: We removed eight proteins that were associated with sex in standard two-tailed, two-sample t-test after correction for multiple comparisons using Benjamini-Hochberg. The final dataset consisted of 1322 proteins (1297 blood and 25 BAL). All analytes were log-transformed for normality before analysis.

### Derivation of data-driven progression signature(s)

Relative fold-changes in the expression levels of individual proteins from the blood and BAL were calculated by dividing the average concentration of each protein in COPD greater decliners by the average concentration in lesser decliners.

Based on proteomic measurements from the COPD participants, we generated optimal progression signatures using EN in tandem with PLSDA for feature selection in the: (a) combined blood and BAL and (b) blood-only datasets. First, the data were randomly sampled without replacement to generate 2000 subsets. To correct for effects of class size imbalances during regularization, we completed resampling at the size of the smallest class. We then performed EN regularization on each of the 2000 subsets. Once regularization was complete, the proteins were subsequently reordered based on their selection frequency throughout the EN iterations and fed in a step-forward manner into the PLSDA algorithm (starting with the protein with the highest selection frequency).

Model performance was evaluated at each step using *k*-fold cross-validation (*k* = 6). The model with the lowest resultant cross-validated error was selected as the optimal classification signature. Alternative minimal signatures were identified as signatures with < 15 features with cross-validated accuracies > 80%. ROC curves were generated based on the classification ability of each PLSDA model. All models were orthogonalized to improve interpretability.

### Comparison of progression signature performance parameters

*Random variants:* To explore the meaningfulness of the optimized signature, we compared its cross-validated performance to that of 2000 random variants. Variant signatures were generated by randomly selecting 52 features from the original dataset. The sixfold cross-validated accuracy was calculated for each random signature. Performance across all variants was compared to the identified signature using a two-tailed, two-sample t-test.

*Cross-validated accuracies:* For quantitative comparisons of cross-validation accuracy across multiple models of interest, we split the data into six groups, iteratively excluded random subsets of 6–7 samples during model calibration, and later used them to test model predictions. The percentage of excluded samples correctly classified in each of the six iterations was used to statistically compare alternative models to the 52-protein signature. We determined statistical significance using a standard one-way ANOVA.

*ROC curves*: To explore the diagnostic ability of binary classifiers, ROC curves were generated from PLSDA models and resultant AUCs were statistically compared using the method outlined by Hanley and McNeil to account for correlation between curves generated from the same cohort^58^. Standard errors were calculated using the Wilcoxon statistic. All reported sensitivities and specificities are generated based on PLSDA model performance, except for the leptin/adiponectin signature, which had metrics stated in the original text^15^.

*Signature enrichment in alternative definitions of progression:* PCA was applied to the 52 proteins identified in the optimal multi-compartment signature (Fig. 2). Participants (n = 45) were labeled using the alternative progression definitions proposed by Anderson et al.^28^: rapid decliners (> 100 mL/year), decliners (20 – 100 mL/year), stable/improvers (< 20 mL/year). All data were mean-centered and variance-scaled prior to analysis. One-way ANOVA with Holm-Šídák’s post hoc test compared participant scores on the first principal component across the groups. Significance was defined as a p-value < 0.05 for all analyses.

### Bioinformatic analysis

*Clustering:* Hierarchical clustering of the 52-feature signature based on blood and BAL proteins was generated with supervised average linkage clustering using Spearman’s correlation coefficient as the distance metric. Samples were colored by progression status.

*Metascape analysis:* Metascape^59^ [https://metascape.org] was used to identify biological processes that were significant and differentially enriched between greater decliners and lesser decliners based on the identified 52-feature signature. PLSDA loadings on LV1 were used to dichotomize proteins between cohorts, such that proteins with positive or negative loadings were increased in lesser decliners or greater decliners, respectively.

*Complement profiles*: PCA was applied to a subset of 22 complement proteins (C1q, C1qBP, C1r, C2, C3d, C3b, C3, C3a, iC3b, C3a des Arg, C4, C4b, C5, C5a, C5-6, C6, C7, C8, C9, Factor B, Factor D, Properdin) measured in the original plasma SOMAscan dataset (outlined above) from greater decliners (n = 14), lesser decliners (n = 31), and a TEPPS reference group (n = 40). Participants were identified as outliers and removed from model if they had a Hotelling’s Reduced T^2^ statistic value > 2, determined via PCA (n = 2). All data were mean-centered and variance scaled prior to analysis. One-way ANOVA with Tukey’s post hoc test compared participant scores across the first principal component. Significance was defined as *p* < 0.05.

### Software summary

Volcano plots and hierarchical clustering were completed using MATLAB (v2017b, MathWorks, Natick, MA). Elastic net was implemented using Glmnet package in MATLAB^60^. We generated PCA and PLSDA models and ROC curves using the PLS toolbox available in MATLAB (v8.2.1, Eigenvector, Mason, WA). All statistics were performed using Prism version 9 (GraphPad Software, San Diego, CA).

## Supplementary Information

Supplementary Information.

## Data Availability

The data that support the findings of this study are available from the corresponding author upon reasonable request and with permission from the SPIROMICS study group.
