# Supplementary material for: A blood and bronchoalveolar lavage protein signature of rapid FEV1 decline in smoking-associated COPD
Source: Sci Rep. 2023 May 22;13:8228. doi: 10.1038/s41598-023-32216-0 (PMC10203309; doi:10.1038/s41598-023-32216-0)
Supplement: Supplementary file 1 — Supplementary Information. [file 41598_2023_32216_MOESM1_ESM.docx]

**SUPPLEMENTAL MATERIALS**

**A blood and bronchoalveolar lavage protein signature of rapid FEV_1_ decline
in smoking-associated COPD**

Katarina M. DiLillo^1^, Katy C. Norman^1^, Christine M. Freeman^2,3,4^, Stephanie A. Christenson^5^,

Neil E. Alexis^6^, Wayne H. Anderson^7^, Igor Z. Barjaktarevic^8^, R. Graham Barr^9^,
Alejandro P. Comellas^10^, Eugene R. Bleecker^11^, Richard C. Boucher^12^, David J. Couper^13^,
Gerard J. Criner^14^, Claire M. Doerschuk^12^, J. Michael Wells^15^, MeiLan K. Han^3^,
Eric A. Hoffman^16^, Nadia N. Hansel^17^, Annette T. Hastie^18^, Robert J. Kaner^19^,
Jerry A. Krishnan^20^, Wassim W. Labaki^3^, Fernando J. Martinez^19^, Deborah A. Meyers^11^,
Wanda K. O’Neal^12^, Victor E. Ortega^21^, Robert Paine, III^22^, Stephen P. Peters^18^,
Prescott G. Woodruff^5^, Christopher B. Cooper^8^, Russell P. Bowler^23^, Jeffrey L. Curtis^3,4,24, +^,

Kelly B. Arnold^1,+,^*, for the SPIROMICS investigators^#^.

^1^ Department of Biomedical Engineering, University of Michigan, Ann Arbor, MI

^2^ Research Service, VA Ann Arbor Healthcare System, Ann Arbor, MI

^3^ Division of Pulmonary & Critical Care Medicine, University of Michigan, Ann Arbor, MI

^4^ Graduate Program in Immunology, University of Michigan, Ann Arbor, MI

^5^ Division of Pulmonary, Critical Care, Allergy and Sleep Medicine, University of California San Francisco, San Francisco, CA

^6^ Center for Environmental Medicine, Asthma, and Lung Biology, University of North Carolina at Chapel Hill, Chapel Hill, NC

^7^ Marsico Lung Institute/Pulmonary and Critical Care Medicine, University of North Carolina at Chapel Hill, Chapel Hill, NC

^8^ Division of Pulmonary and Critical Care Medicine, Department of Medicine, University of California Los Angeles, Los Angeles, CA

^9^ Department of Medicine, Columbia University Medical Center, New York, NY

^10^ Division of Pulmonary, Critical Care and Occupational Medicine, University of Iowa, Iowa City, IA

^11^ Division of Genetics, Genomics and Precision Medicine, University of Arizona Health Sciences, Tucson, AZ

^12^ Marsico Lung Institute/Cystic Fibrosis Research Center, Department of Medicine, University of North Carolina at Chapel Hill, Chapel Hill, NC

^13^ Collaborative Studies Coordinating Center, Department of Biostatistics, University of North Carolina at Chapel Hill, Chapel Hill, NC

^14^ Department of Thoracic Medicine and Surgery, Temple University, Philadelphia, PA

^15^ Department of Medicine, University of Alabama at Birmingham, Birmingham, AL

^16^ Department of Radiology, University of Iowa, Iowa City, IA

^17^ Division of Pulmonary and Critical Care Medicine, Johns Hopkins University School of Medicine, Baltimore, MD

^18^ Department of Internal Medicine, Wake Forest School of Medicine, Atrium Health, Wake Forest Baptist, Winston Salem, NC

^19^ Department of Medicine, Weill Cornell Medical Center, New York, NY

^20^ Division of Pulmonary, Critical Care, Sleep and Allergy, University of Illinois at Chicago, Chicago, IL

^21^ Department of Internal Medicine, Division of Respiratory Medicine, Mayo Clinic, Scottsdale, AZ

^22^ Division of Respiratory, Critical Care, and Occupational Pulmonary Medicine, University of Utah, Salt Lake City, UT

^23^ Division of Pulmonary and Critical Care, National Jewish Health, Denver, CO

^24^ Medical Service, VA Ann Arbor Healthcare System, Ann Arbor, MI

^#^ A list of authors and their affiliations appears at the end of the paper

^+^ Co-senior authors

* Corresponding author

**Supplementary Table S1. Baseline characteristics of COPD cases and TEPPS reference group**

|  |  | **TEPPS^§^**  (N=40) | **Greater Decliners^**^** (N=14) | **Lesser Decliners** (N=31) | **P-Value** |  |
| --- | --- | --- | --- | --- | --- | --- |
| **Age*** |  | 59.3 (± 8.67) | 64.2 (± 6.24) | 63.1 (± 8.41) | 0.068 |  |
| **Currently Smoking*** |  | 13 (32.5%) | 5 (35.7%) | 10 (32.3%) | 0.97 |  |
| **BMI*** |  | 29.3 (± 5.01) | 27.9 (± 3.67) | 27.8 (± 5.43) | 0.41 |  |
| **Sex** (Male) |  | **17 (42.5%)** | **12 (85.7%)** | **17 (54.8%)** | **0.02^†^** |  |
| **Race**  (White/Other) |  | 29/11 (72.5%) | 12/2 (85.7%) | 25/6 (80.6%) | 0.52 |  |
| **ICS use*** (yes) |  | **2 (5.0%)** | **3 (21.4%)** | **14 (45.2%)** | **0.0003^‡^** |  |
| **FEV_1_*** (% predicted) |  | **100.4 (± 13.1)** | **84.2 (± 13.1)** | **71.1 (± 17.7)** | **< 0.0001****^†‡^** |  |
| **FEV_1_/FVC*** |  | **0.78 (± 0.04)** | **0.60 (****± 0.08)** | **0.57(± 0.10)** | **< 0.0001^†‡^** |  |
| **FEV_­1_*** (L) |  | **2.89 (± 0.69)** | **2.63 (± 0.60)** | **2.11 (± 0.66)** | **< 0.0001^‡^** |  |
| **Visit 5 FEV_1_** (L) |  | **2.74 (± 0.77)** | **1.97 (± 0.67)** | **1.93 (± 0.68)** | **< 0.0001^†‡^** |  |
| **Time from baseline**  **to Visit 5** (yrs) |  | 6.18 (± 0.93) | 6.25 (± 0.76) | 6.33 (± 0.91) | 0.77 |  |
| **Time from baseline to**  **bronchoscopy** (months) |  | **12.5 (±11.1)** | **20.4 (± 10.0)** | **20.3 (± 12.5)** | **0.0107^‡^** |  |
| **∆FEV_1_** (mL/yr) |  | **-25.7 (± 47.5)** | **-104.6 (± 32.0)** | **-28.8 (±21.5)** | **< 0.0001^†^** |  |
| One-way ANOVA with Tukey’s post hoc test or chi-squared test were used to determine significance.  * Demographic information from baseline visit (Visit 1)  † Significant difference between TEPPS and greater decliners  ‡ Significant difference between TEPPS and lesser decliners  § Tobacco-exposed people with preserved spirometry (TEPPS); n=2 removed prior to analysis due to identification as outliers (see methods)  ** Defined as annualized decline in FEV_1_ ≥ 70 mL/year (see Methods) | | | | | | |

**Supplemental Table S2. Demographics of study group compared to full SPIROMICS bronchoscopy sub-study cohort**

|  | **Study Group**  (N=85) | **Full Bronchoscopy Cohort**  (N=188) | **P-Value** |
| --- | --- | --- | --- |
| **Age*** | 61.5 (± 8.41) | 60.1 (± 8.89) | 0.24 |
| **Currently Smoking*** | 28 (32.9%) | 81 (43.1%) | 0.11 |
| **BMI*** | 28.5 (± 5.01) | 28.5 (± 4.97) | 0.99 |
| **Sex** (Male) | 46 (54.1%) | 104 (55.3%) | 0.85 |
| **Race**  (White/Other) | 66/19 (77.6%) | 131/57 (69.7%) | 0.17 |
| **ICS use*** (yes) | 19 (22.4%) | 37 (19.7%) | 0.61 |
| **FEV_1_*** (% predicted) | 87.0 (± 19.9) | 88.1 (± 19.1) | 0.67 |
| **FEV_1_/FVC*** | 0.67 (± 0.12) | 0.68 (± 0.12) | 0.75 |
| **FEV_­1_*** (L) | 2.56 (± 0.75) | 2.59 (± 0.76) | 0.75 |
| **Visit 5 FEV_1_** (L) | 2.32 (± 0.82) | 2.33 (± 0.80) | 0.87 |
| **Time from baseline**  **to Visit 5** (yrs) | 6.25 (± 0.89) | 6.28 (± 0.97) | 0.81 |
| **Time from baseline to**  **bronchoscopy** (months) | 16.6 (± 12.0) | 15.2 (± 12.4) | 0.37 |
| **∆FEV_1_** (mL/yr) | -39.8 (± 47.0) | -34.4 (± 51.7) | 0.46 |

Unpaired two-tailed t-test or chi-squared test were used to determine significance.

**
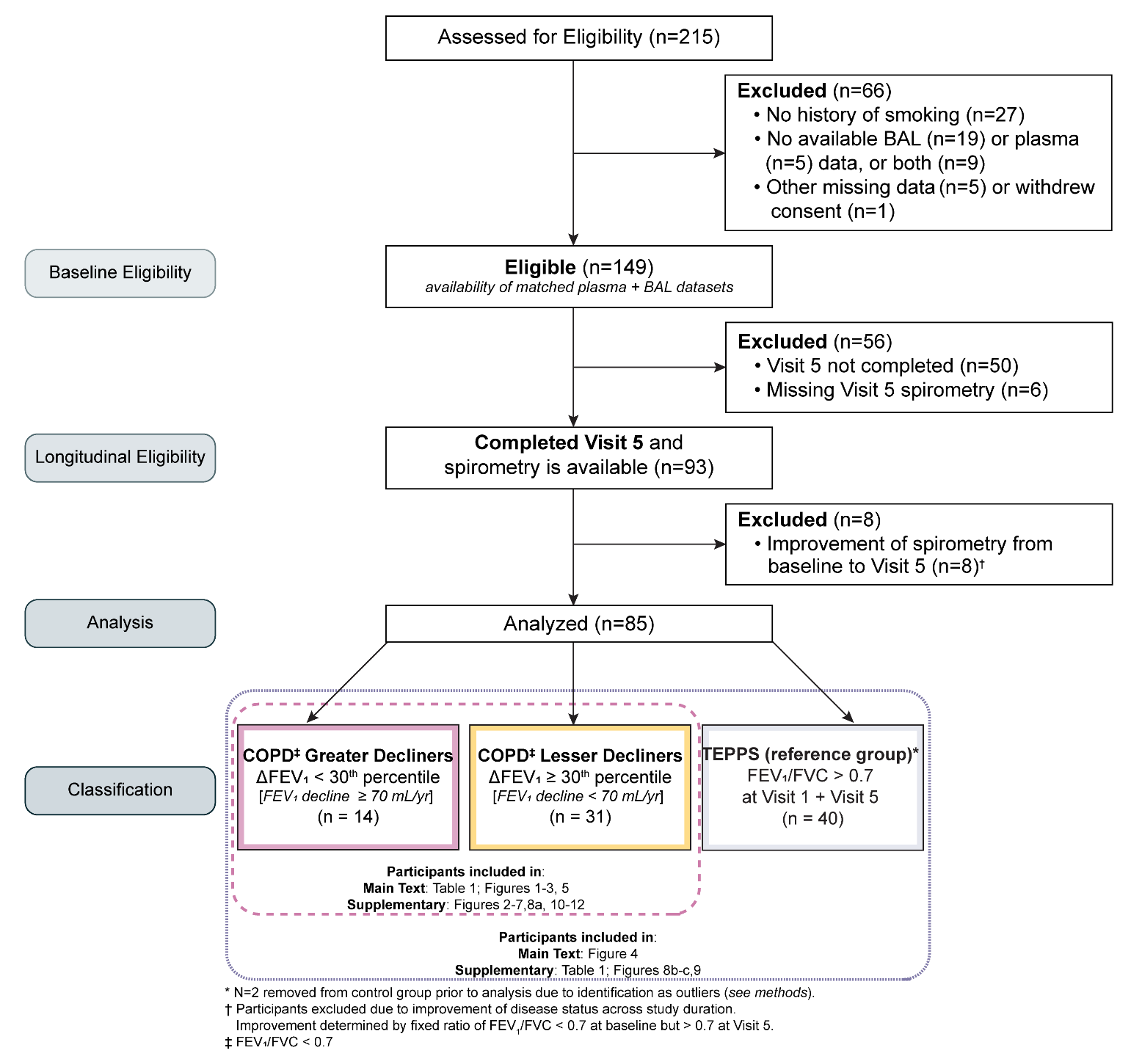
**

**Supplementary Figure S1.** Schematic illustrating the inclusion criteria and patient breakdown of participants with a history of smoking from SPIROMICS I bronchoscopy sub-study (n=215) included in analysis.

**
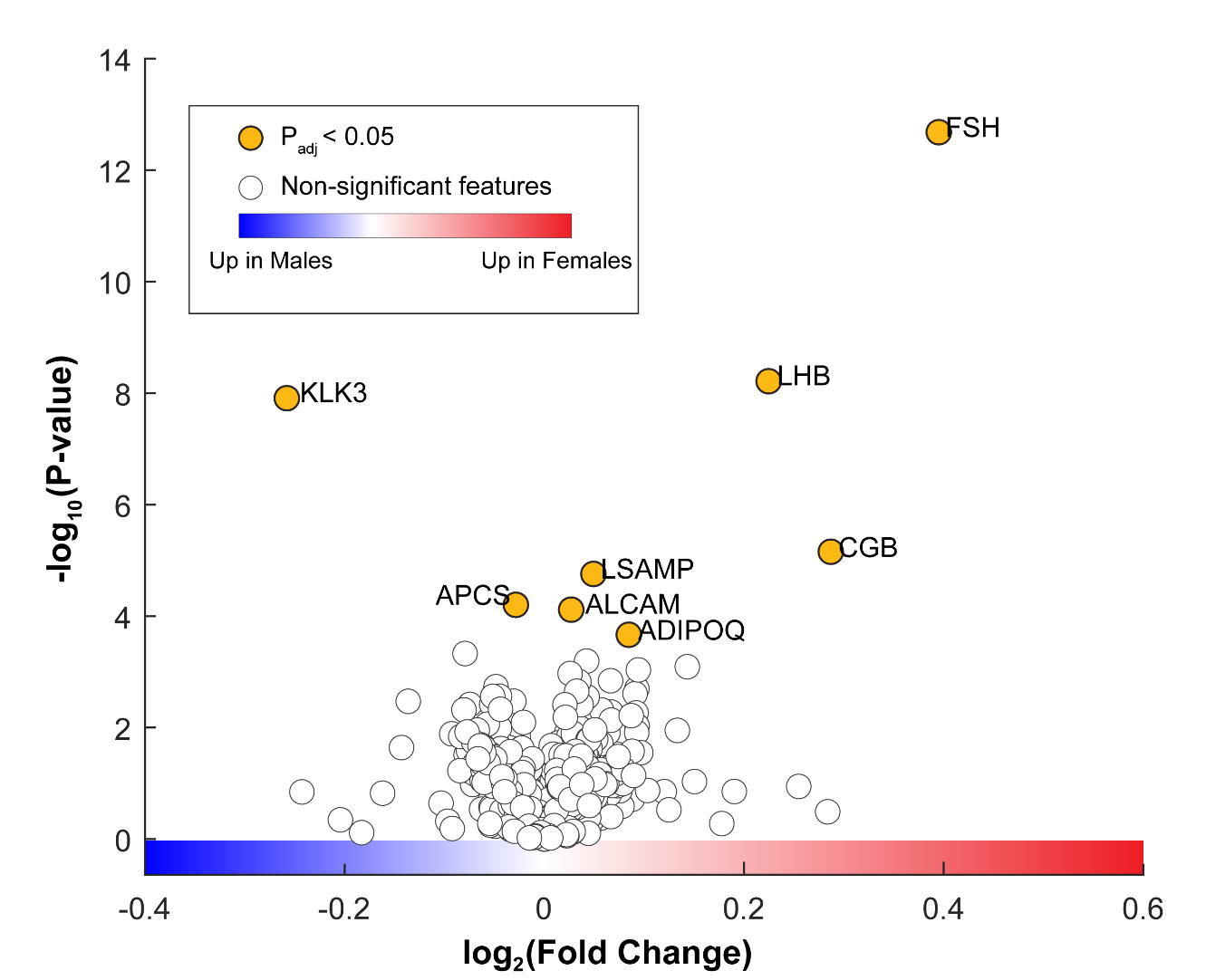
**

**Supplementary Figure S2. Differential expression of cross-compartment proteins between sexes.** Volcano plot of blood and BAL proteins (two-sampled, two tailed t-test). Yellow markers represent proteins which have p-value < 0.05 after correction for multiple comparison using Benjamini-Hochberg false discovery rate (FDR) (α = 0.05).

**
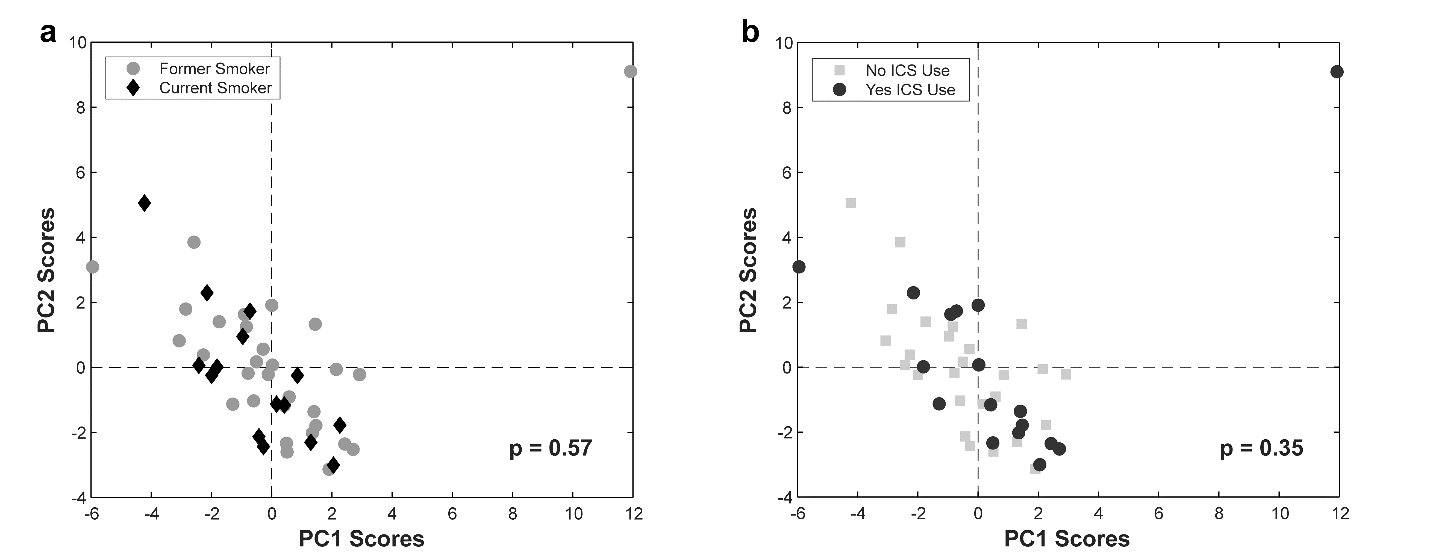
**

**Supplementary Figure S3. 52-ft cross-compartment signature is not influenced by participants’ smoking status or ICS use.** PCA was completed with the 52 (blood and BAL) proteins identified in the multi-compartment progression signature (Figure 2; n=45). Participants (n=45) are classified into two groups using self-reported (a) baseline smoking status (current/ former) or (b) ICS use within three months of the baseline visit (yes/no). P-values reported from a permutation test (n = 2000 permutations) between groups’ mean scores across PC1 and PC2.

**Supplementary Figure S4. The 52-feature elastic net-identified signature outperforms random variants of equal size.** Comparison of optimal model CV accuracy (98.4%) to average performance of 1000 random variant signatures, generated by selecting randomized feature sets (proteins) from the original dataset (1322 proteins) at a size equal to our optimal signature (52 features; two-tailed two-sample t-test; ****p < 0.0001).

**
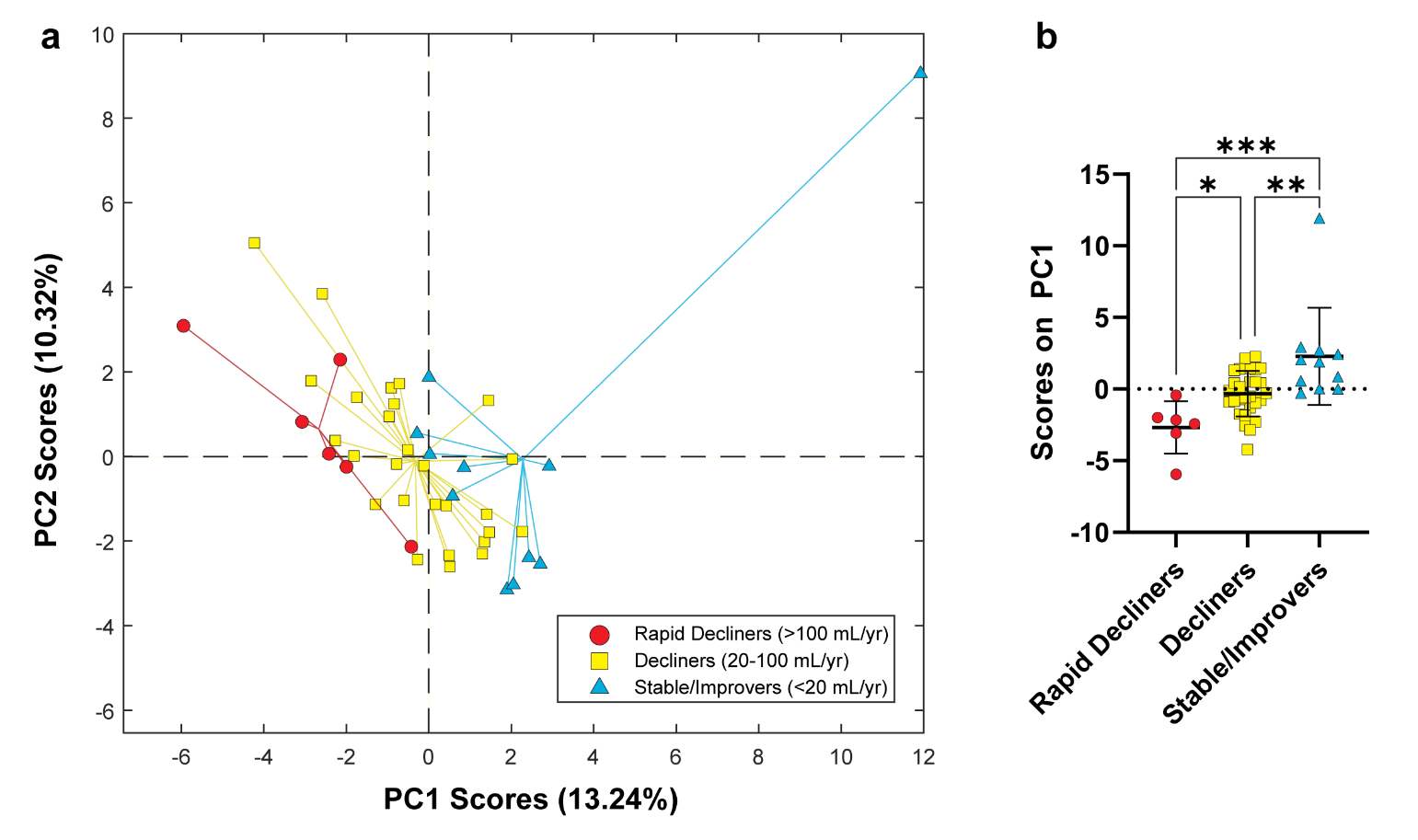
**

**Supplementary Figure S5. Multi-compartment progression signature is significantly enriched in an alternative, more stringent definition of rapid lung function decline.** (a) PCA completed with the 52 (blood and BAL) proteins identified in the multi-compartment progression signature (Figure 2). Participants (n=45) are classified into 3 groups using the alternative progression definitions proposed by Anderson et al.^28^: rapid decliners (circles; >100 mL/year), decliners (squares; 20 – 100 mL/year), stable/improvers (triangles; < 20 mL/year). (b) Comparisons of participant scores across PC1 show significant enrichment of signature in rapid decliners by this definition, compared to decliners and stable/improvers (one-way ANOVA with Holm-Šídák's multiple comparisons test; *p < 0.05, ** p < 0.01, *** p < 0.001).


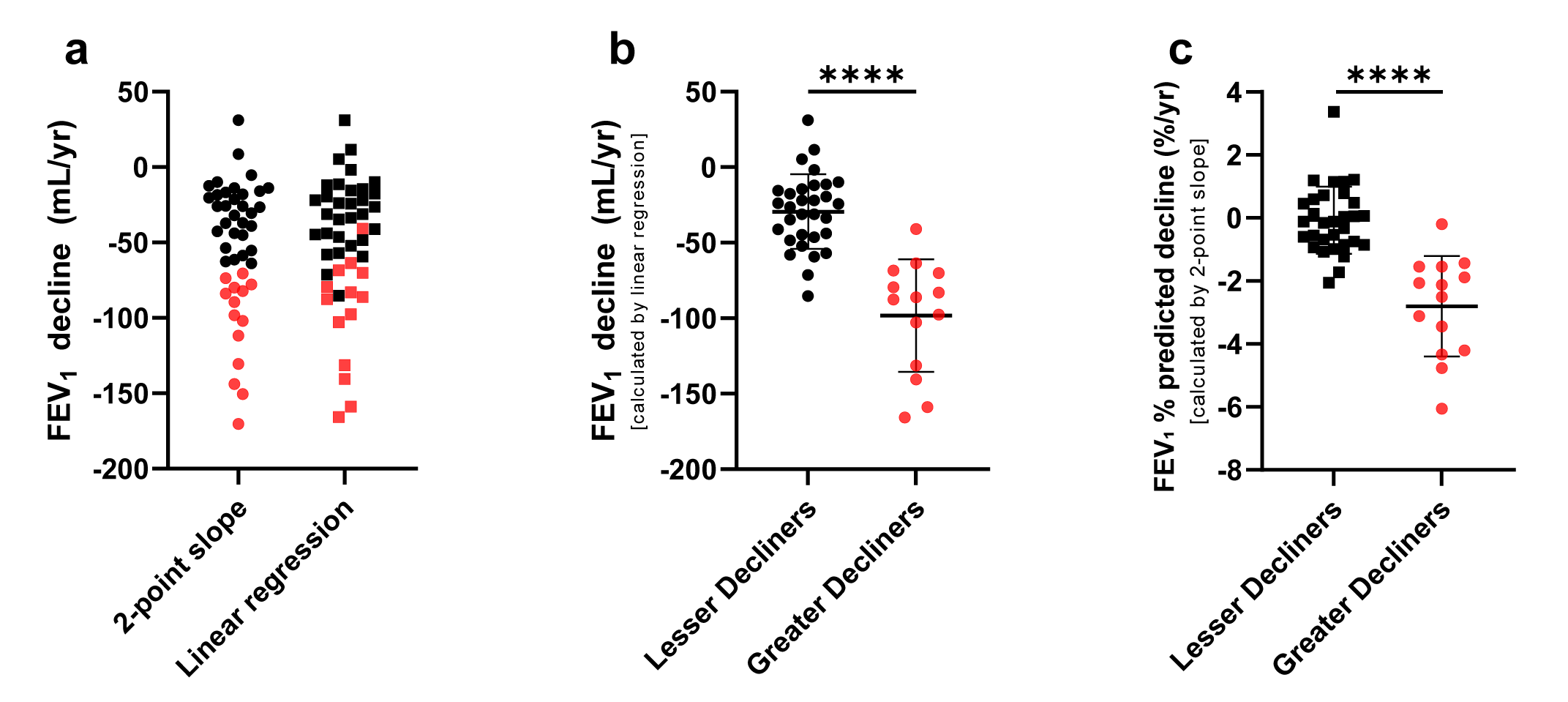


**Supplementary Figure S6. Classifications are not largely affected by alternative calculation approaches.** (a) Visualization of the impact of calculating FEV_1_ decline using 2 data points (Visit 1 and Visit 5) versus all available longitudinal spirometry. FEV_1_ declines using 2 data points (left) were calculated using the 2-point slope equation: $\frac{V5 FEV_{1}-V1 FEV_{1}}{Time from V5 to V1}$ . The multiple point estimate (right) was defined as the slope ($\beta_{1}$) of the linear regression equation $FEV_{1}=\beta_{0}+\beta_{1}\left( Time since V1 \right)$ for each participant. (b) Plot of FEV_1_ (mL/yr) values generated from the linear regression approach, grouped into greater or lesser decliners based on their 2-point slope evaluations scheme. A two-sample t-test suggests that using only Visit 1 and 5 data is sufficient to capture more complex progression trends. (c) Plot of lung function decline calculated using FEV_1_ % predicted at Visit 1 and 5 exhibit similar distributions as the original calculations, which use absolute FEV_1_ (two-sample t-test; ****p < 0.0001). Red points denote participants classified as greater decliners in all plots as per the 2-point evaluation calculations completed with absolute FEV_1_. For all estimates, the change in time was calculated using the visit dates for each participant; calculations assumed a fixed-length year equal to 365.2425 days.

**
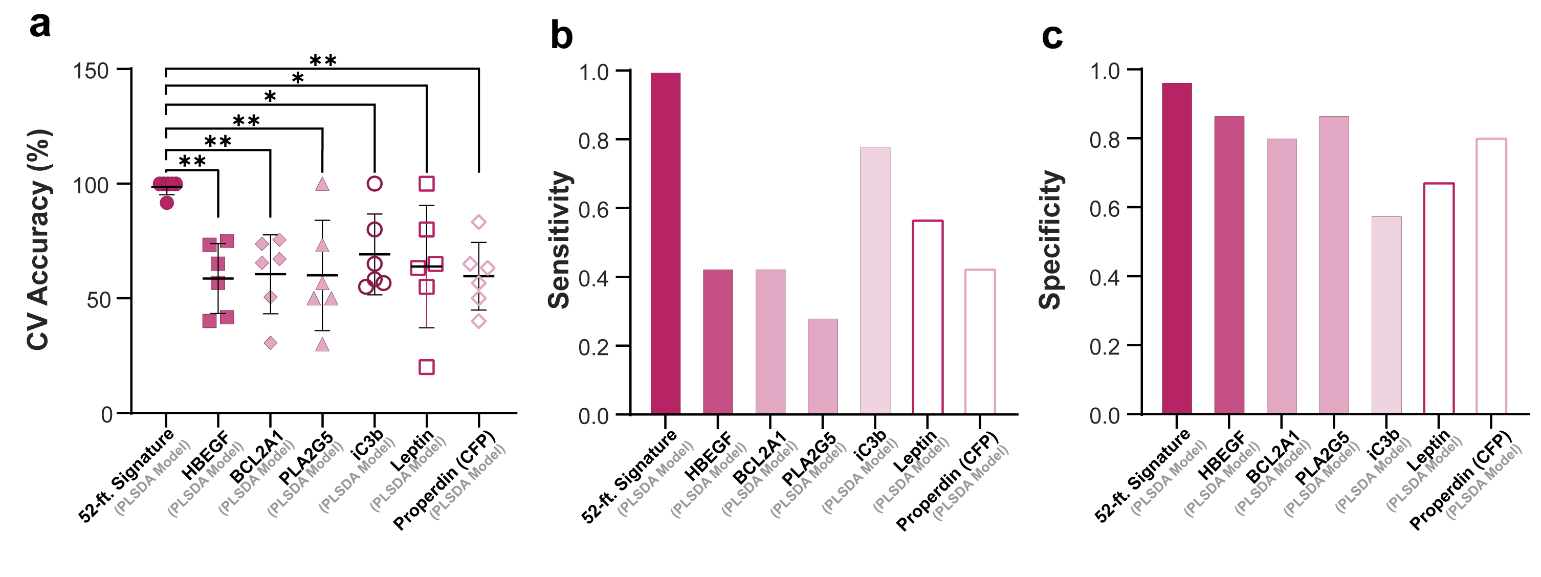
**

**Supplementary Figure S7. The 52-feature significantly outperforms analyses based on individual proteins.** (a) Comparison of 6-fold cross-validation (CV) accuracies, (b) sensitivities, and (c) specificities between optimized data-driven signature and the top six individually identified proteins in Fig. 1b. One-way ANOVA with Dunnett’s post hoc test; *p<0.05, **p<0.01.

**
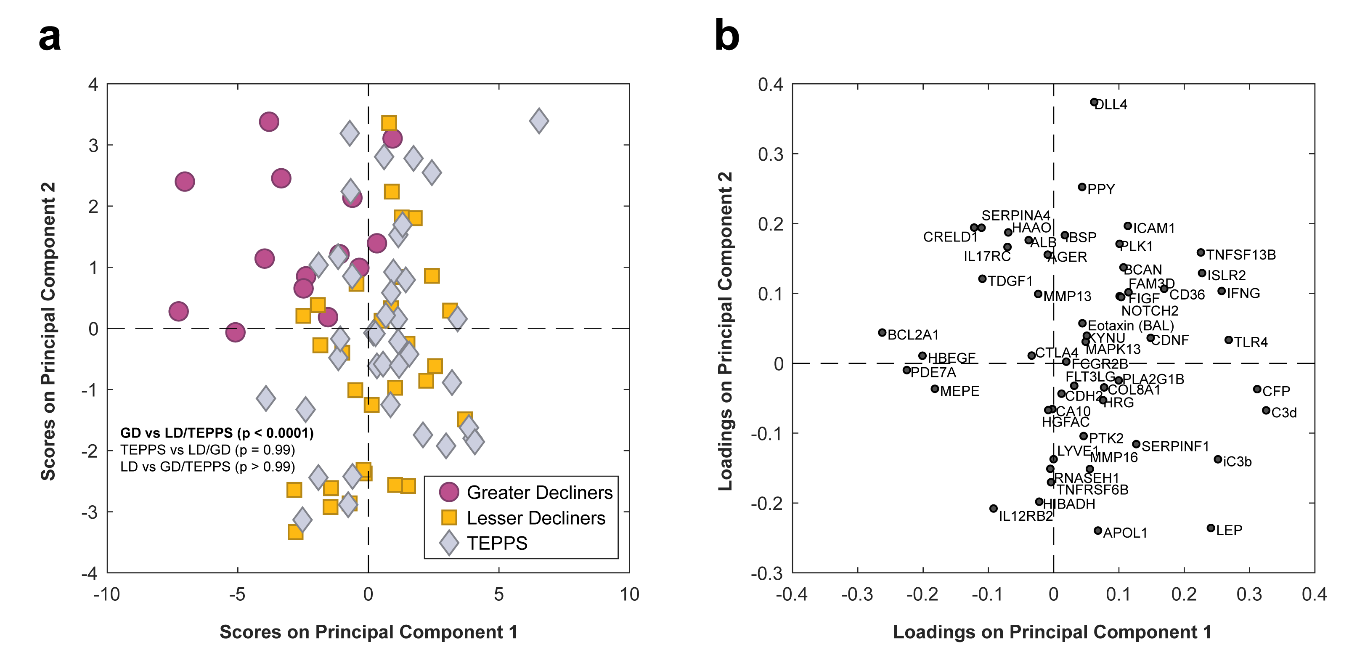
**

**Supplementary Figure S8. 52-feature cross-compartment signature can differentiate TEPPS from Greater Decliners but not Lesser Decliners.** (a) PCA scores plot generated with the 52 signature-identified cross-compartment proteins of greater decliners (circles), lesser decliners (squares), and a reference group of tobacco-exposed people with preserved spirometry (TEPPS) (diamonds). First two principal components (PCs) capture 17% of the variance in the dataset. P-values reported from a permutation test (n = 2000 permutations) between groups’ mean scores across PC1 and PC2. (b) Protein loadings across PC1 and PC2.

**
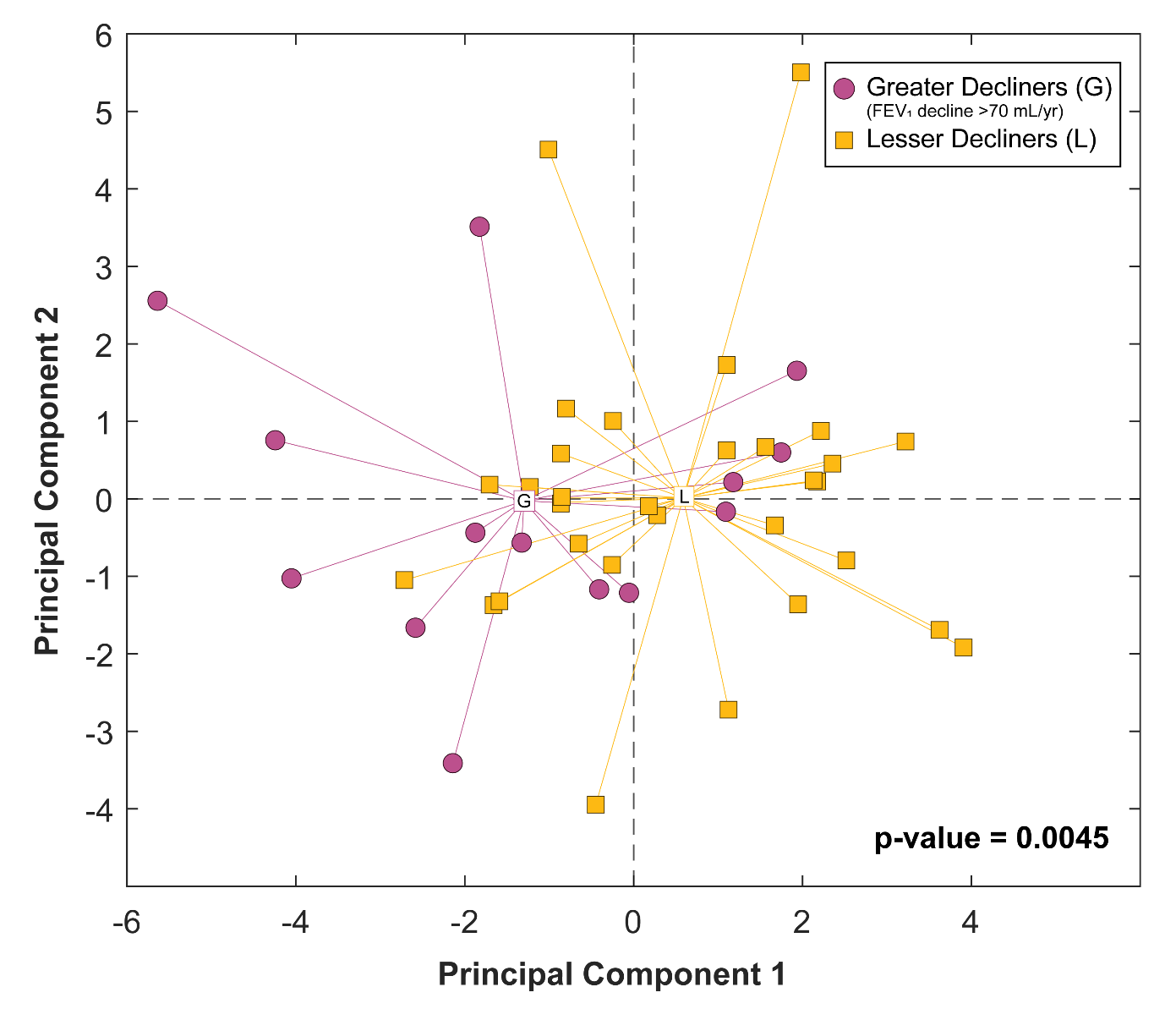
**

**Supplementary Figure S9. At baseline, greater decliners have unique complement profiles.** PCA of all complement proteins measured in plasma (including C1q, C1qBP, C1r, C2, C3d, C3b, C3, C3a, iC3b, C3a des Arg, C4, C4b, C5, C5a, C5-6, C6, C7, C8, C9, Factor B, Factor D, Properdin). The first two principal components collectively capture 34.8% of total variance. P-value shown for two sample, two-tailed t-test of PC1 scores across groups.

**
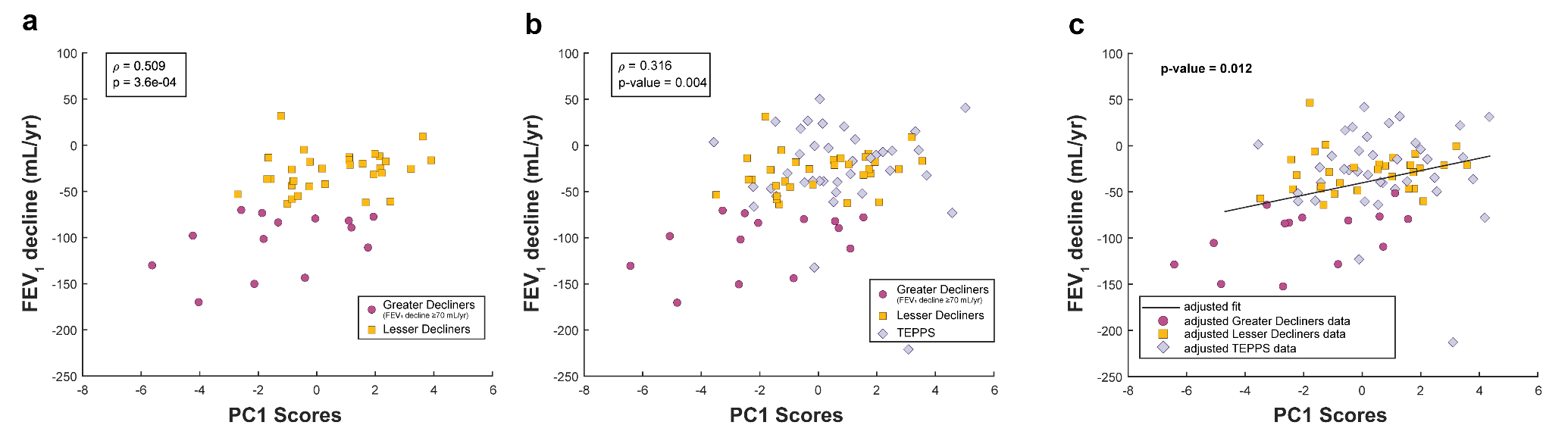
**

**Supplementary Figure S10. PC1 scores from the complement-specific PCA highly correlate with decline in FEV_1_ (mL/yr).** This analysis uses the PC1 scores from Supplementary Figure 6. (a) Pearson correlation of PC1 scores and FEV_1_ mL/ year in a model with greater decliners and lesser decliners alone and (b) with the addition of a reference group of tobacco smoke-exposed persons with preserved spirometry (TEPPS). (c) Observed relationship between complement protein profiles on PC1 and FEV_1_ decline remains significant after adjusting for clinical covariates (p-value shown for linear regression adjusted for age, race, height, sex, baseline FEV_1_% predicted, smoking status, ICS use within three months of baseline visit, and pack-years).

**
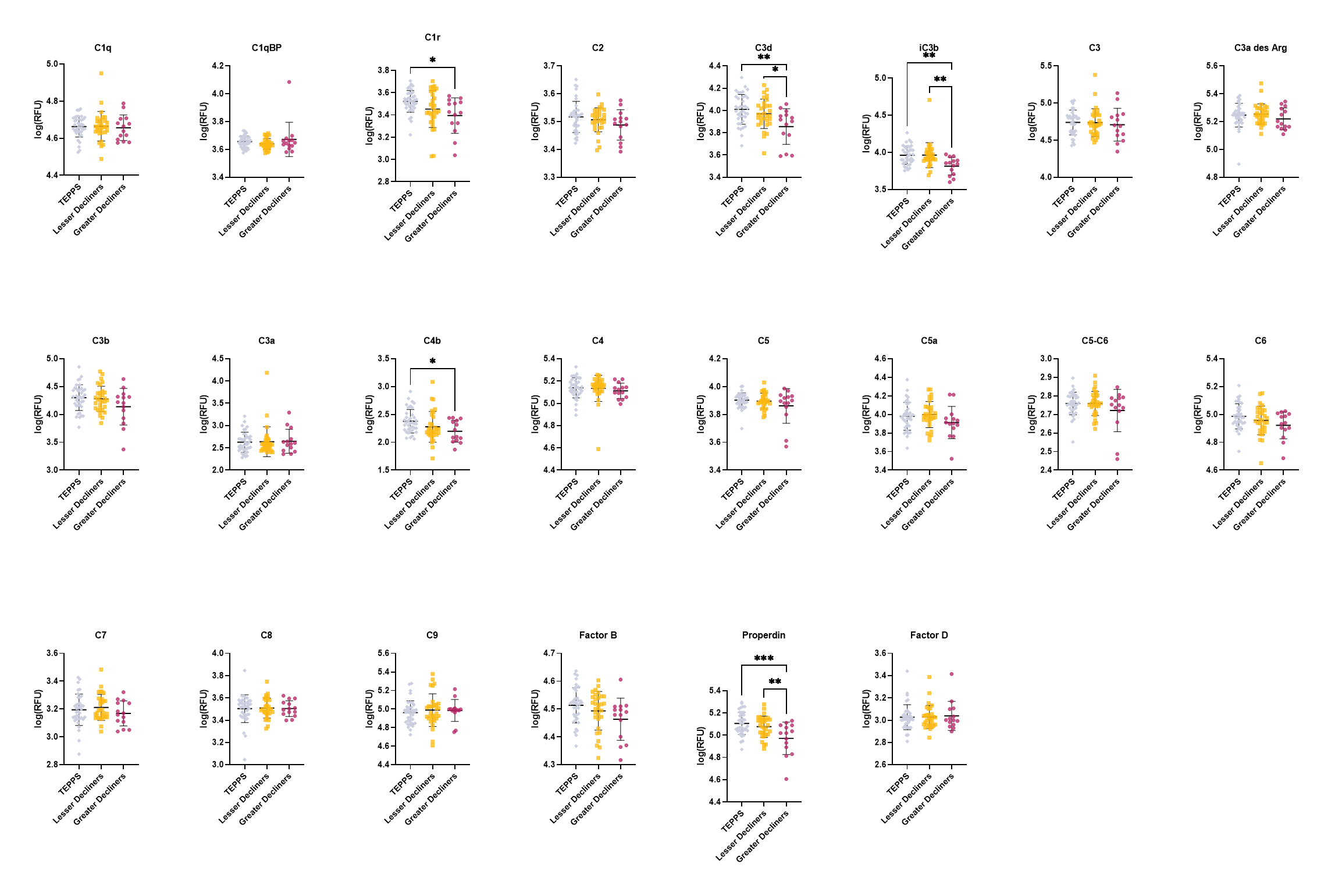
**

**Supplementary Figure S11. Univariate comparison of complement cascade proteins in blood.** Participant groups are as in the legend to Supplementary Figure 7. Individual levels of complement-associated proteins in greater decliners, lesser decliners, and TEPPS reference group (one-way ANOVA with Tukey’s post hoc test; *p<0.05, **p<0.01, ***p < 0.001).

**
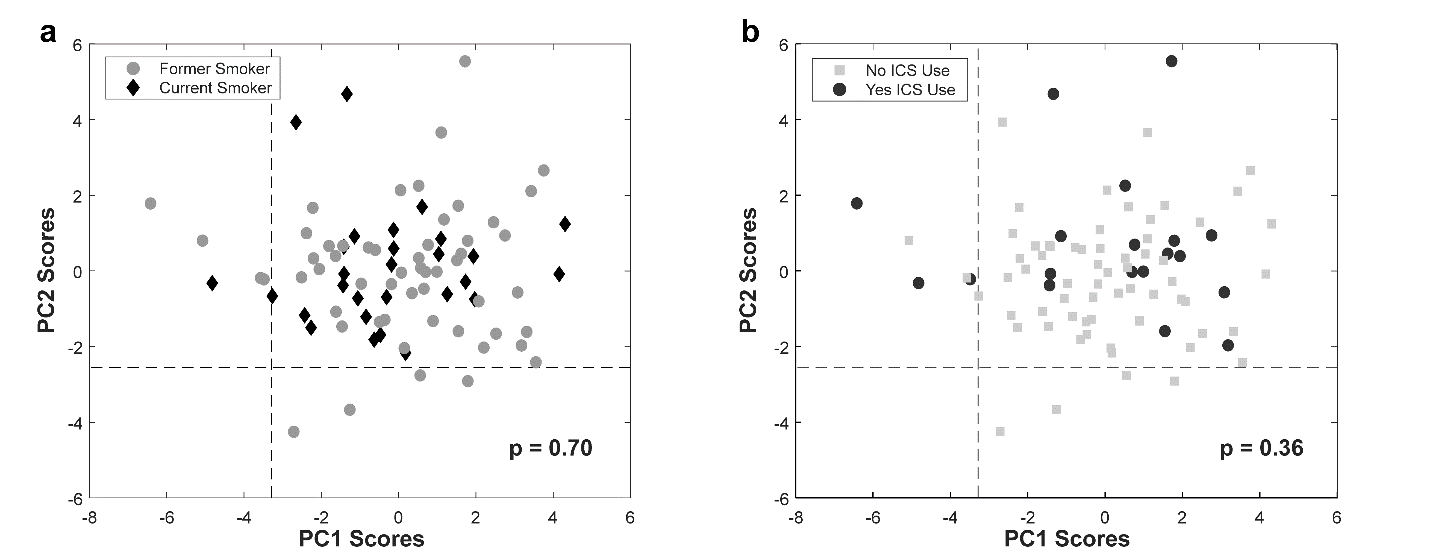
**

**Supplementary Figure S12. Complement profiles are not influenced by participants’ smoking status or ICS use.** Complement PCA profile (Figure 4; n=83) classified into two groups using self-reported (a) baseline smoking status (current/ former) or (b) ICS use within three months of the baseline visit (yes/no). P-values reported from a permutation test (n = 2000 permutations) between groups’ mean scores across PC1 and PC2.

**
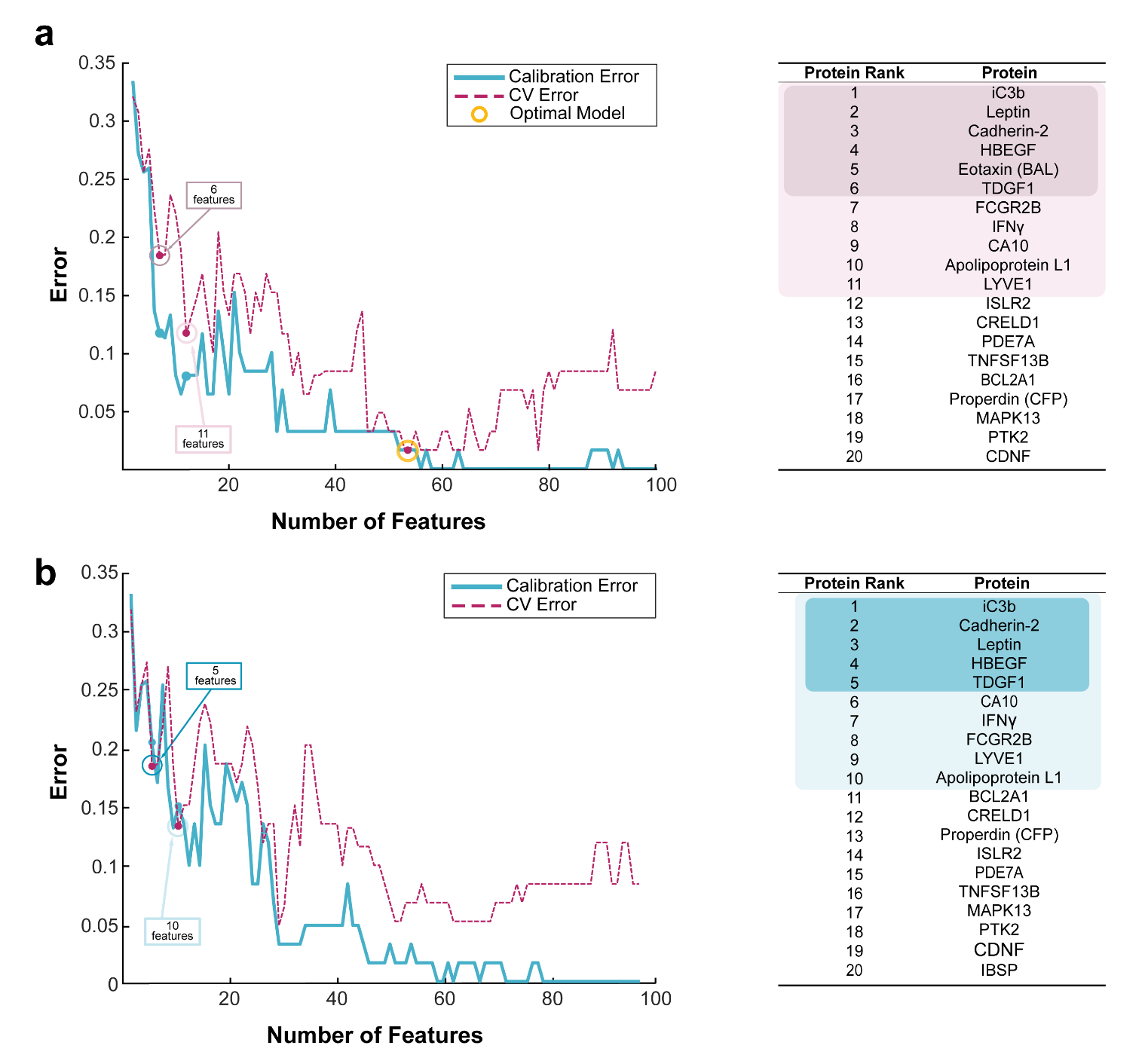
**

**Supplementary Figure S13. Stepwise PLSDA identified minimal signatures with strong cross-validated performance.** Plot of calibration and CV errors from stepwise PLSDA models for (a) cross-compartment and (b) blood-only datasets. Smaller models that separated groups with statistically comparable CV accuracies to the 52-feature models are highlighted with call out boxes (11-feature signature: 88.4%; 6-feature signature: 81.6%; 10-feature signature: 86.8%; 5-feature signature: 81.6%). Accompanying lists on the right depict the order of proteins added in models based on Elastic Net resampling selection frequency of proteins (1 = most frequently selected). Highlighted proteins denote those in the identified minimal signatures displayed in the plots.

**
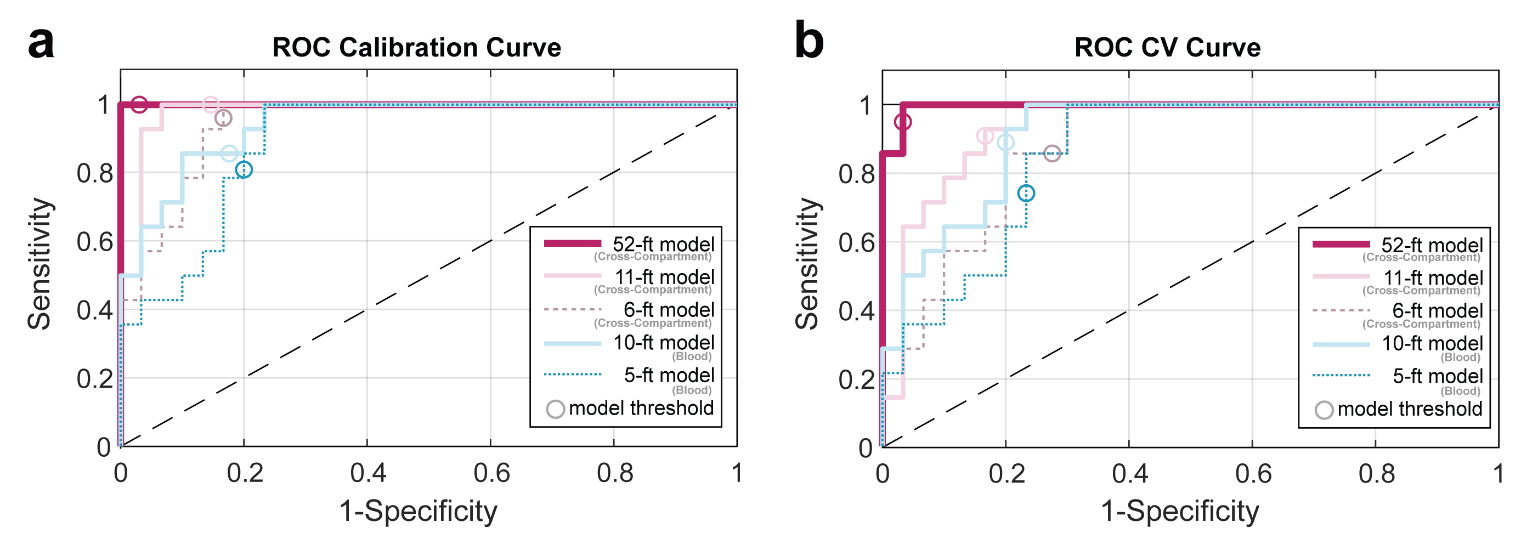
**

**Supplementary Figure S14. ROC curves from PLSDA models generated from minimal signatures**. ROC curves from cross-compartment and blood minimal signatures generated from (a) calibration and (b) CV PLSDA models.

**
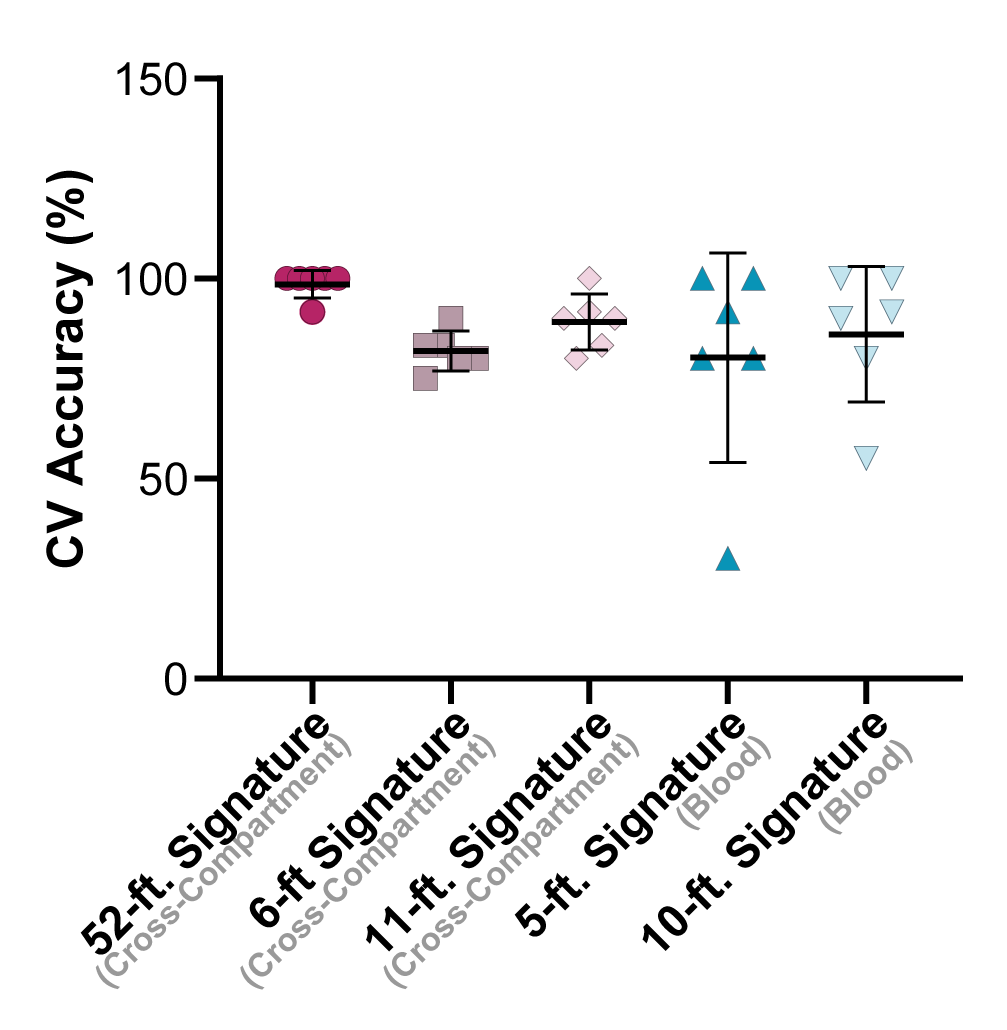
**

**Supplementary Figure S15. Cross-validation accuracies do not significantly vary between optimal model and smaller variants.** Comparisons of 6-fold CV accuracies between models (one-way ANOVA with Dunnett’s post hoc test).
